# High-resolution X-ray scanning with a diffuse Huffman-patterned probe to reduce radiation damage

**DOI:** 10.1107/S1600577525002127

**Published:** 2025-04-09

**Authors:** Alaleh Aminzadeh, Andrew M. Kingston, Lindon Roberts, David M. Paganin, Timothy C. Petersen, Imants D. Svalbe

**Affiliations:** a Department of Materials Physics, Research School of Physics Australian National University Australia; b CTLab: National Centre for Micro-Computed Tomography Australian National University Australia; c School of Mathematics and Statistics University of Sydney Australia; d School of Physics and Astronomy Monash University Australia; e Monash Centre for Electron Microscopy Monash University Australia; Tohoku University, Japan

**Keywords:** Huffman sequences, diffuse probe, X-ray imaging, gray-level masks

## Abstract

Scanning objects with a tightly focused beam (of photons or electrons for example) can provide high-resolution images. However, rapid deposition of energy into a small area can damage tissues in organic samples or may rearrange the chemical structure or physical properties of inorganic materials. Scanning an object with a broad, or diffuse, beam can deliver an equivalent probe energy but spread it over a much wider footprint. However, typically the imaging resolution is proportional to the probe diameter and a diffuse probe sacrifices resolution. Here we propose a method to achieve ‘high resolution’ imaging (in the sense that resolution is smaller than the probe diameter) using a diffuse probe. We achieve this by encoding a pattern onto the probe and employing a decoding step to recover a tight delta-like impulse response. Huffman sequences, by design, have the optimal delta-like autocorrelation for aperiodic (non-cyclic) convolution and are well conditioned. Here we adapt 1D Huffman sequences to design 2D Huffman-like discrete arrays as diffuse imaging probes that have spatially broad, relatively thin, uniform intensity profiles and have excellent aperiodic autocorrelation metrics. Examples of broad shaped diffuse beams were developed for the case of X-ray imaging. A variety of masks were fabricated by the deposition of finely structured layers of tantalum on a silicon oxide wafer. The layers form a pattern of discrete pixels that modify the shape of an incident uniform beam of low-energy X-rays as it passes through the mask. The intensity profiles of the X-ray beams after transmission through these masks were validated, first by acquiring direct-detector X-ray images of the masks, and second by raster scanning a pinhole over each mask pattern, pixel-by-pixel, collecting ‘bucket’ signals as applied in traditional ghost imaging. The masks were then used to raster scan the shaped X-ray beam over several simple binary and ‘gray’ test objects, again producing bucket signals, from which sharp reconstructed object images were obtained by deconvolving their bucket images.

## Abstract 2

This paper introduces high-resolution imaging using diffuse probes, which allow for lower energy deposition per unit area per unit time, by encoding Huffman-like patterns onto them, enabling a tighter impulse response. The approach, demonstrated in X-ray imaging, involves using specially fabricated masks to shape the probe and recover sharp object images through robust deconvolution.

## 1. Introduction

High-resolution scanning X-ray imaging typically requires high-fidelity beam shaping to create sharp probes (Yan *et al.*, 2014 ▸; Bajt *et al.*, 2018 ▸), concentrating the radiation that is raster scanned over the specimen. The same is true for atomic-resolution scanning transmission electron imaging, where it is advantageous to further remove probe aberrations in dedicated post-processing to achieve the highest resolution (Chen *et al.*, 2021 ▸). Irrespective of resolution, beam shaping can be also useful for patterning the scattered radiation to sensitively measure strain (Zeltmann *et al.*, 2020 ▸) or efficiently map crystal orientations (Hong *et al.*, 2021 ▸). An alternative to raster scanning is to transmit a known ensemble of broad yet structured probes through a specimen, to be computationally reconstructed through ‘ghost imaging’ (Erkmen & Shapiro, 2010 ▸; Pelliccia *et al.*, 2016 ▸; Yu *et al.*, 2016 ▸). Here we consider combining these ideas to produce a broad patterned probe that encodes the sample through scanning, and seek to recover sharp images through correlative reconstruction akin to ghost image recovery.

Energy deposition has paramount importance in the X-ray imaging of soft-tissues or nano-structured materials. Mitigating beam damage is likewise crucial for atomic resolution structural determination in cryo-electron microscopy, and low electron dose imaging continues to be developed for beam-sensitive materials such as metal organic frameworks, zeolites and organic perovskites. The motivation for imaging with a diffuse beam is to significantly reduce the rate of energy deposited per unit area of the test object. For example, a uniformly intense beam having an area of 10 × 10 pixels reduces the rate of incident energy deposition per unit area by 100 times relative to that from an equivalent probe focused to be one pixel wide. Deconvolving the diffuse beam to single-pixel width reduces the radiation damage whilst preserving the spatial resolution and signal-to-noise ratio.

A closely related technique for structuring the incident beam is the use of coded apertures that employ ‘binary’ 2D masks comprising elements which have either 100% transmission or are fully opaque (Fenimore & Cannon, 1978 ▸). When combined with a spatially resolved or pixelated camera, coded apertures are useful in contexts where lensing is not possible, as a generalization of pinhole imaging [see the review by Cieślak *et al.* (2016 ▸)]. For X-ray imaging, coded apertures are generally specialized binary (2D) masks designed to improve spatial resolution and enhance contrast in a range of applications, including transmission X-ray imaging, phase contrast imaging (Olivo & Speller, 2007 ▸), compressive X-ray tomosynthesis (Cuadros *et al.*, 2015 ▸), 3D diffraction contrast imaging (Gürsoy *et al.*, 2025 ▸) and high-energy astrophysics (Braga, 2020 ▸).

Common designs of coded apertures include uniformly redundant arrays (URAs) (Fenimore & Cannon, 1978 ▸), modified URAs (MURAs) (Haboub *et al.*, 2014 ▸) and Hadamard patterns (Pinilla *et al.*, 2018 ▸). As Huffman patterns are designed for aperiodic convolution (unlike URAs, MURAs and Hadamard patterns), Huffman probes can be used to scan regions of arbitrary size.

This paper addresses the conceptual and experimental design, fabrication and application of 2D masks with the primary motivation to reduce damage to specimens being scanned during X-ray imaging. A spatially broad beam of uniform intensity incident onto these masks transmits an equally broad but discretely shaped pattern of illumination. Each digital pattern is designed to have an autocorrelation that is approximately a delta function. Using this intensity pattern as an X-ray probe to scan a test object produces a blurred image of the object as it is convolved by the pattern of the mask. The inverse problem of decoding the pattern encoded by the mask is straightforward due to the delta-like autocorrelation property of the mask. We have called this process ‘high-resolution’ scanning with a diffuse probe since the imaging resolution achievable is smaller than the probe diameter. While initially motivated by reducing dose rate (and thus sample damage) given synchrotron radiation, we note that this concept can also improve the imaging resolution given any radiation that is not easily focused, *e.g.* lab-based X-rays, or neutrons.

In this work, the intensity of a broad beam from a synchrotron was shaped, at a discrete pixellated level, to form a diffuse probe. The broad probe shape is designed to be deconvolved to yield a point-spread function with single-pixel resolution. Shaping a probe requires controlled changes to the intensity transmitted through each pixel of the mask used for beam shaping. The near parallel and near monochromatic X-rays from a synchrotron means the probe intensity could be controlled by transmitting the broad beam through a mask comprising different thicknesses and/or different materials for each individual pixel of the mask. This experiment could also be done using incident optical photons, lab-source X-rays, electrons or neutrons if precise control of the beam absorption can be achieved. This method is applicable wherever it proves difficult to maintain the focus of an incident beam down to the desired pixel width. We also note that here single-pixel (or bucket) signals were collected and used to reconstruct images of the scanned objects, but this approach is not essential to the method. The images of the scanned test samples here show absorption contrast, but could also have been configured to display phase contrast, near- or far-field scatter, fluorescence or other imaging modalities.

The remainder of the paper proceeds as follows. Section 2 reviews the theory behind the design of diffuse, delta-like pixellated masks that transmit a narrow range of stepped intensities using beams that project onto areas comprising hundreds to thousands of pixels. The mask design was inspired by earlier work on 1D aperiodic sequences by Huffman (Huffman, 1962 ▸). Section 3 describes the design and the challenging techniques developed here to fabricate Huffman-like masks; this includes both binary masks suited for use with X-rays with an energy of around 20 keV, and quaternary masks designed for use with an X-ray energy of 12.4 keV. The latter masks were built as multiple uniform layers of tantalum deposited in discrete patterns on a silicon oxide wafer. Validation of the as-fabricated patterns, which was achieved by directly imaging the X-ray beam transmitted by each mask, is presented in Section 4. The experimental synchrotron-beamline setup, images of the fabricated test objects, and the experimental procedures (including post-processing analysis) are expained in Section 5. Section 6 presents experimental results obtained using a uniform beam of synchrotron-generated X-rays that was shaped by transmission through these masks. Several simple binary objects and more complex ‘gray’ test objects were scanned with these diffuse beams to produce bucket images from which images were reconstructed that closely match the test objects. We discuss the experiment results and their implications in Section 7. Section 8 summarizes our findings and discusses future practical imaging applications for broad Huffman-like masks. Additional relevant information on the properties of Huffman sequences, the design and testing of compressed Huffman-like arrays and a more detailed overview of the fabrication design and wafer production process necessary to make the masks used in the X-ray imaging experiment can be found in the supporting information.

## 2. Numerical design of broad, diffuse X-ray probes with delta-like auto-correlation

Scanning probes acquire images of test objects that are blurred by the spread and shape of the scanning probe.

To reduce the impact of the local radiation dose rate of the incident beam, the probe area could be made broader or more diffuse. A broader beam generally means poorer spatial resolution. However, a diffuse probe shape that is encoded with a pattern that has an autocorrelation closely approximating a delta function can circumvent this issue; we refer to such probes as having *delta-like autocorrelation*. The object image that was blurred by the shape of the probe can be restored by a deconvolution using the known shape of the probe. Probes that have delta-like autocorrelation are well conditioned and ensure that the inverse deblurring problem is robust to measurement noise. The method may be viewed as a form of coded aperture imaging (Cieślak *et al.*, 2016 ▸), in which the coding and decoding arrays are identical to one another.

This section describes how to encode such sequences into suitable shapes for use as scanned X-ray probes. Much attention has been given in the literature to the many and varied periodic sequences that are delta-like under circular convolution or correlation (so-called *periodic arrays*) (Chu, 1972 ▸; Schmidt, 2016 ▸; Petersen *et al.*, 2024 ▸). However, these sequences are impractical when the probe field-of-view (FOV) limits the object size. We usually need to scan objects larger in size than the probe FOV. In this situation, the probe performs non-circular (or aperiodic) convolution or correlation. We hence seek to encode (or pattern) the probe shape using sequences that possess delta-like *aperiodic* autocorrelation. The Huffman sequences described in the next section are, by design, optimal for aperiodic convolution.

### 2.1. Huffman sequences and 2D Huffman arrays

In 1962, in a pioneering paper, Huffman (1962 ▸) defined what constitutes the optimal possible delta-like form for any aperiodic autocorrelation and then constructed quite special examples of sequences, *H*_*L*_, for any length *L*, that met that goal.

Following that work, several different types of integer, real and complex Huffman sequences have been found. These sequences, specified here as 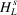 for distinct sequence types *s*, all have in common the following unique property under *aperiodic* autocorrelation (⊗),
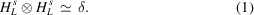
Here, δ is the Kronecker delta function,
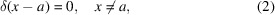
with *x* and *a* being array coordinates. So-called ‘perfect-sequences’, *S*_*L*_, where 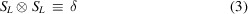
under *periodic* autocorrelation conditions, were well known long before Huffman’s work (Schmidt, 2016 ▸). However, those sequences do not satisfy equation (1) when used in aperiodic operations.

Building on Huffman’s paper, Hunt & Ackroyd (1980 ▸) discovered integer-valued Huffman sequences (of length *L* = 4*n* − 1) with entries that turn out to be derived from the Lucas/Fibonacci series. An example of their sequence type is 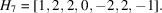
The aperiodic autocorrelation of *H*_7_ is 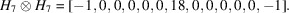
An aperiodic autocorrelation is *optimally delta-like* [following equation (1)] when all of the off-peak autocorrelation values are zero bar the unavoidably non-zero left and right end correlation values. The magnitudes of the end values are kept as small as possible relative to the autocorrelation peak, which should be as large as possible. For *H*_7_, those values are −1 and 18, respectively. ‘Perfect-periodic’ sequences, for example Legendre *L*_7_ = [0, 1, 1, −1, 1, −1, −1], are not delta-like (for *L*_7_, the aperiodic autocorrelation peak is 6 with many non-zero off-peak values ranging from +1 to −2). Thus, 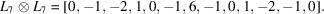
The range of and spacing between the values in the elements of 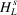 has critical practical implications for the arrays fabricated in our experiment, for example to construct the 32 × 32 array. The 1D Lucas/Fibonacci sequence *H*_31_ has signed integer values that range between ±754. The integer range for these Huffman sequences grows exponentially with length *L*,
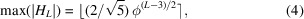
where ϕ is the golden ratio, 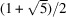, and 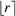 denotes the integer round operation on real value *r*. The ranges fixed by equation (4) motivate the compression of Huffman sequence values, as covered in Section 2.3.

In general, any *canonical* Huffman sequence of length *L* has optimal aperiodic autocorrelation of length 2*L* − 1 with the form 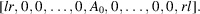
The peak of the auto-correlation value at zero shift is *A*_0_, whilst the (unavoidable) end terms *rl* = *lr* arise from the product of the (non-zero) leftmost and rightmost elements of the sequence 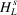, denoted here as *l* and *r*, respectively. Then equation (1) is the closest possible approximation to an aperiodic δ, especially for integer sequences when *rl* = ±1 (|*l*| need not match |*r*| for all Huffman sequence types).

The elements of Huffman sequence *H*_*L*_ comprise *L* signed values that, under the convolution operator, act as a discrete, broadband filter. To be delta-like, the bandwidth of 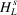 as a filter has to have a near-constant response over all *L* spatial frequencies in order for equation (1) to hold. Further examples of integer Huffman sequences and their properties are given in the supporting information, together with a detailed description of their Fourier spectra.

Huffman derived the necessary and sufficient criteria to compute canonical sequences 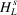 in terms of a complex polynomial *P*(*z*): in the complex plane, any root/zero *z*_*l*_ of *P*(*z*) must lie on a circle of radius either *R* or 1/*R* with phase angle arg(*z*_l_) = 2π*l*/(*L* − 1). See Huffman (1962 ▸) and related work by Ackroyd (1977 ▸) and Ojeda & Tacconi (1994 ▸).

A polynomial with *L* coefficients *c*_*l*_, where 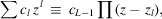
can be represented as a discrete Fourier transform 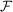 when evaluated on the unit circle by setting *z* = 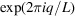, where the integer *q* lies in the range 0 ≤ *q* ≤ (*L* − 1). Given a set of roots *z*_*l*_, an inverse Fourier transform 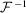 can thus efficiently compute and sort the coefficients *c*_*l*_.

Using Huffman’s criteria for roots placement, the *q*th Fourier coefficient 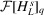 of any canonical Huffman sequence hence can be calculated from 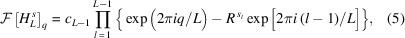
where *s*_*l*_ ∈ {−1, +1} is any chosen set of signs. While the elements of 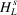 are sensitive to the 2^*L*−1^ choices for *s*_*l*_, each choice produces an identical canonical autocorrelation function. How equation (5) adheres to Huffman’s original construction is explained in the supporting information, as is the straightforward restriction to real-valued 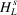. By careful choice of *R*, the growth of 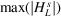 with increasing *L* [such as in equation (4)] can be suppressed, yet the values are generally non-integer. Therefore the dynamic range of 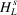 still increases for larger *L*.

Huffman sequences that have a compact range of element values and close-to-zero off-peak aperiodic autocorrelation elements are particularly suited for X-ray imaging applications. We call sequences that are optimized to adhere closely to equation (1) ‘Huffman-like’.

### 2.2. Encoding X-ray probes with 2D Huffman arrays

A patterned scanning probe based on a 2D Huffman array *H* can be constructed from 1D *H*^*s*^ sequences using an outer product *, such that *H* = *H*^*s*^ * *H*^*s*^ also obeys the desired property given in equation (1) (Svalbe *et al.*, 2020 ▸). Suppose a 2D X-ray probe has been encoded by a mask defined by *H*. For an object of interest *O* convolved (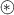) with *H*, a sharp image of *O* can be obtained by cross-correlation via the property in equation (1),
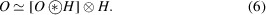
Thus far we have described how to generate 2D Huffman arrays with the optimal aperiodic autocorrelation property. The delta-like autocorrelation means that we can robustly compute a high-resolution image (of arbitrary size due to the aperiodic probe properties) by scanning a test object with a broad beam patterned by these arrays. In the context of X-ray imaging, a beam with a wider footprint is often preferable, to diffuse the X-ray dose more effectively. However, as shown by equation (4), the range of array values grows rapidly with increased array size. The fabrication of masks that are able to transmit X-rays with a large range of discrete intensity values (or a wide range of discrete phase shifts in the wavelength) is intrinsically difficult. Practical construction concerns require we place strong limits on the value range of transmitted X-ray intensities (or phase shifts).

Experimentally, a physical attenuation used to moderate X-ray intensities can only comprise non-negative variations in intensity. We propose a method to achieve this in the following section (Section 2.2.1). To be practical from a fabrication point of view, the mask should also be restricted to providing only a few different intensities comprising clearly separated uniform steps. This is achieved by compressing the dynamic range of Huffman sequences to manageable levels whilst still retaining their Huffman properties.

Fully compressing the range of signed integer values in Huffman sequences would produce, as its ultimate limit, a binary sequence. However, as shown by White *et al.* (1977 ▸), no real binary sequence can have the canonical aperiodic auto-correlation property. Complex-valued Huffman sequences can be designed to have near-unity magnitude, but they all require multi-valued phase. For this reason, our Huffman-like sequences (even with integer values constrained to ±3) will, under aperiodic convolution, outperform all binary sequences (including perfect periodic sequences) of the same length.

The quality metrics used to quantify array performance are detailed in Section 2.2.2. Having established these metrics, we then present two different design approaches in Section 2.3 to ensure that equation (1) is maintained, as closely as is possible, after compressing the gray-level range of Huffman arrays.

#### 2.2.1. Non-negative 2D X-ray masks to imprint Huffman arrays

Huffman arrays necessarily comprise signed elements, while X-ray intensities are non-negative. Huffman arrays can nonetheless be realized using pairs of absorptive X-ray masks, provided that the decorrelation method equation (6) is generalized. We split the signed values of the Huffman-like array *H* into two positive-definite parts as ‘Huffman masks’ – one comprising the positively signed elements, *P* = 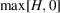, and another containing the magnitudes of the negative elements, *N* = max[−*H*, 0], such that 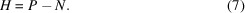
If a signal *S*_*T*_ encodes an object *O* of interest with a Huffman-like array via a linear relation such as *S*_*T*_ = 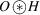, this total signal can be decomposed as the difference between two successive measurements *S*_*p*_ and *S*_*n*_ using the Huffman mask arrays *P* and *N*, respectively, 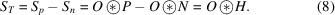
The desired object signal *O* can then be de-correlated by cross-correlating *S*_*T*_ with the delta-correlated *H*.

In our experiment, the X-ray beam, after being shaped by the array, is to be raster scanned across the object being imaged. Both *P* and *N* masks need to be applied separately. We arranged each *P* and *N* mask in a 2 × 2 block, *i.e.*
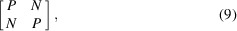
that we will denote inline as [*P*, *N*/*N*, *P*]. This layout permits the *P* and *N* probes to be scanned separately across the object, along either the horizontal or vertical scan axes. This redundant mask arrangement also provides multiple ways to combine *P* and *N* bucket images to form the bucket image from any mask *H*.

Having solved how, in practice, to deal with negative mask intensities, the next problem is to constrain the number of intensity levels that each *P* and *N* mask transmits. More and more finely spaced transmission levels impose significantly tighter demands on mask fabrication. The range of Huffman mask values can be compressed, but practical truncation or rounding of the compressed levels rapidly reduces their essential delta-like autocorrelation property.

We chose to limit the design range of our Huffman-like arrays to ±3. The *P* and *N* arrays then have four uniformly spaced steps [0, 1, 2, 3], where 0 means the lowest and 3 means the highest transmitted intensity. An intensity range larger than ±3 would prove, in practice, difficult to fabricate for X-rays. A key aim of this experiment is to keep the broad beam intensity profile as wide and as uniform as possible to minimize the rate of energy deposition.

For larger sized arrays, we found that limiting array values to ±2 produced a mostly binary ±1 result that cannot capture the broad central peak of intensities that is typical of classic integer Huffman sequences. On the other hand, each array element that has value ±3 increases the size of the autocorrelation peak *A*_0_ by 9 (and each ±2 value adds increases of 4). In contrast, array values of ±1 add just 1 to *A*_0_. Sequences with all values ±1 are known to not satisfy equation (1), which is essential for our imaging context (excluding the special case of area-weighted binary masks presented in Section 2.3.3).

The relevant metrics used to guide the range-compression process are described in the next section.

#### 2.2.2. Array delta-correlation quality metrics

Here we present metrics to quantify the desirable properties of the diffuse probe patterns. All the following metrics were monitored and simultaneously optimized when compressing Huffman array element values. The metric definitions are shown below for sums over the 2D arrays used here, but apply equally for 1D and *n*D arrays, where the sums are taken over all array elements.

Our primary aim is to minimize the adverse effects of the probe causing local heating or radiation damage to the scanned object. To achieve this, the diffuse X-ray probe should transmit close-to-uniform intensities over the entire area of the mask to maximally spread the dose. This property can be quantified for an *L* × *L* array *H*_*L*_ by:

RMS. The root mean square of intensities transmitted by an array is 
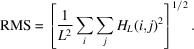
RMS values of 1 correspond to the same uniform (positive) intensity transmitted through each array element.

MAV. The mean absolute value is 
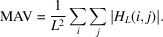
MAV values of 1 (for uniform transmission across the mask) are preferred.

An array with a strongly compressed range of integer intensities may also set many array elements to zero, reducing the RMS and MAV values closer to 1. However, opaque regions in an X-ray mask do not transmit specimen information and so diminish the imaging efficiency. A further useful quality measure is then:

*f*^*z*^. The fraction *f*^*z*^ of zero intensities in the array pattern, 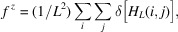
where δ(η) = 1 when η = 0 and 0 otherwise. Arrays with *f*^*z*^ ≃ 0 (with any zero elements located peripherally rather than centrally) are preferred. This metric can also be applied to the (2*L* − 1) × (2*L* − 1) autocorrelation array. Then more zeros are preferred and *f*^*z*^ ≃ 1.

In this work we use three different measures to quantify how closely the autocorrelation of each array is delta-like to satisfy equation (6). Here the array peak autocorrelation value is *A*_0_ and the off-peak autocorrelation values are *A*_*i*,*j*_, for shifts −*L* ≤ (*i*, *j*) ≤ *L*, for (*i*, *j*) not both zero.

*M*^*f*^. The ‘merit factor’ is the square of the autocorrelation peak value divided by the sum of all squared off-peak autocorrelation values. Here larger values are preferred, with 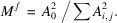


*R*^0^. The ‘peak to side-lobe’ is the ratio of the peak autocorrelation value to the largest of all the off-peak autocorrelation absolute values. Larger values are preferred, with 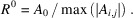


*d*^*F*^. ‘Spectral flatness’ has several possible definitions, one being the ratio of the geometric to arithmetic mean of the Fourier magnitudes (as used in acoustics). Here we use 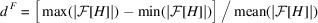
as this measure proved to be more sensitive to the extremes of variation in the magnitude of the Huffman-like array Fourier transform coefficients 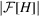. Values closer to zero are preferred.

Well conditioned arrays are preferable since they are more robust to noise and inversion is a stable process. To measure how well conditioned the arrays are (especially after range compression), we use the singular value decomposition (SVD) of the array, taken under zero-padded aperiodic boundary conditions, with singular values stored in the set σ(*H*_*L*_).

κ. The condition number is defined as the ratio of largest to smallest singular values, *i.e.*
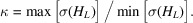
Note that κ ≥ 1 and values closer to 1 are preferred.

The metrics corresponding to four 11 × 11 examples are presented in Table 1 ▸. These demonstrate that, under aperiodic convolution, Huffman arrays are indeed very strongly delta-like. This remains true for their Huffman-like versions, even after their integer element values have been compressed (from ±36) to a more practical range (±3). In contrast, the Barker binary array shows poorer autocorrelation metrics relative to the Huffman-like array, as does the Legendre binary ‘perfect’ array (the latter being designed to excel in the periodic domain). The autocorrelation metric values *R*^0^, *M*^*f*^ generally *increase* with array size (as more element values are added); however, it becomes more difficult to keep *d*^*F*^, the Fourier flatness metric, closer to zero.

### 2.3. Compressing Huffman arrays as practical masks to encode diffuse probes

The dynamic range of Huffman array elements can, to some extent, be controlled by the choice of the chosen phase distribution. Similarly the radius and sign choices [*R* and *s*_*l*_, respectively, in equation (5)] can reduce extrema in Huffman-like arrays. To improve upon such initial choices, systematic procedures are developed here to further compress Huffman arrays while optimizing the metrics of Section 2.2.2. We refer to compressed integer-valued Huffman element values as Huffman-like arrays having a small range of (signed) integer image gray levels.

#### 2.3.1. Iterative optimization of compressed Huffman array element values

For integer Huffman sequences, the range of values usually grows with length, as given by equation (4) for the example of the Lucas/Fibonacci sequences. Recall that for *H*_31_ the integers range over ±754 and that the value range for a 2D array is the square of the 1D range. For practical mask fabrication, where constructing as few levels as possible is preferred, strong range compression is mandated, especially for arrays with side-lengths *L* > 7.

Scaling each Huffman element value by the same constant preserves all autocorrelation metrics. The non-linear operation of integer rounding to enact range-compression on the other hand alters the otherwise flat power spectrum, which can in turn degrade all correlation metrics. To counter this, we iteratively optimize the metrics of Section 2 by initially down-scaling the Huffman array such that the smallest element values retain unit-magnitude (rather than rounding such element values to zero). All element values are scaled down by a real number *v* (by no more than a factor of two) in fine steps while the metrics of Section 2.1 are monitored after integer rounding. When an optimal value of *v* is determined, ‘dithering’ is used by adding an *L* × *L* array of zero-mean white noise, with maximum magnitude less than 1/2. The Section 2 metrics are monitored after integer rounding, again to find an optimal random perturbation. The process of down-scaling and optimizing arrays is iterated until the desired dynamic range (of ±3) is reached.

The sequence compression process is driven by monitoring for continual improvement in the metrics *R*^0^, *M*^*f*^ and *d*^*F*^, usually in that order of priority (as randomly structured sequences can be spectrally flat). When conflict in metric gains occurs, such as *R*^0^ decreasing while *M*^*f*^ improves, the compression step size is reduced and metric *d*^*F*^ is given more priority.

The final optimization step randomly cycles through each array value (for example, choosing those array elements with value −2) and perturbs a small random sample of those elements by changes of ±1, again monitoring for changes in the Section 2 metrics to fine-tune the array.

After random perturbation of 2D arrays, transpose symmetry is restored by taking (and rounding) the mean of the array and its transpose after each optimization step. However, while our preference is to preserve this symmetry, it is possible that the transpose operation produces an array with an inferior set of metrics. In this case the asymmetric array is retained.

Whilst outer products of a canonical Huffman sequence of length *L* are suitable for building small *L* × *L* arrays, for longer lengths (*i.e.L* > 100), compression of very large dynamic ranges [as given by equation (4)] can yield erratic noise-like sequences that are difficult to optimize. Airy functions can instead be used as flatter-ranged impulse-like sequences, or ‘chirps’. Portions of these 1D chirps are used to construct larger Huffman-like 2D arrays via an outer-product (an example of a range-compressed 120 × 120 array is given in the supporting information).

#### 2.3.2. Hybrid Monte Carlo optimization of Huffman array element values

Bernasconi (1987 ▸) showed that maximizing the merit factor *M*^*f*^ of binary sequences is akin to minimizing the energy of spins in a 1D Ising model. Bernasconi’s 1D Ising approach for constructing Barker-like binary sequences (Borwein *et al.*, 2004 ▸) (for example, *B*_11_ as given earlier) using Metropolis–Hasting Monte Carlo simulations (Bernasconi, 1987 ▸) needs to be generalized here to construct integer-valued arrays with autocorrelation adhering to equation (6).

To implement Markov steps, with reference to equation (5), we choose a starting radius *R* to define an initial Huffman sequence, and a random set of signs {*s*_1_, *s*_2_,…, *s*_*L*−1_} is fixed. A sign *s*_*c*_ is then randomly chosen and randomly incremented/decremented by a small (sub-integer) random step size, sampling from a uniform distribution, such that the magnitudes of the complex roots are now defined by the non-canonical set 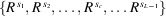 (where |*s*_*c*_| now differs from unity). After quantizing the associated Huffman sequence elements (or array for 2D), this random non-integer *s*_*c*_ change is then accepted outright if the merit factor improves or conditionally accepted according to an exponential distribution of the weighted square difference.

Our method for iteratively evolving the complex root radii was tested here for Barker-like unit-magnitude 1D sequences, while also mimicking simulated annealing by linearly varying the square-difference weight for the change in merit factor, in addition to the step size (to optimize the ratio of accept/reject moves). All known Barker sequences were efficiently found from these simulations in this work, in addition to all Barker-like sequences with largest possible merit factor (tested up to length 20).

This generalization of Bernasconi’s energy-based Monte Carlo algorithm (Bernasconi, 1987 ▸) only optimizes the merit factor *M*^*f*^, yet we need to optimize all metrics of Section 2.2.2 in order to achieve the desired property in equation (1) for Huffman-like arrays. With further adaption here, hybrid reverse Monte Carlo (McGreevy & Pusztai, 1988 ▸; Opletal *et al.*, 2002 ▸) (HRMC) is ideally suited for this purpose. While originally designed to minimize energy and maximize consistency with experimental diffraction measurements in atomic systems, including larger scale porosity constraints (Petersen *et al.*, 2007 ▸; Opletal *et al.*, 2013 ▸), HRMC optimization can be used in radically different contexts [*cf*. a recent urban design study of flooding (Balaian *et al.*, 2024 ▸)].

For such optimization of Huffman arrays in 2D, the quantization we have chosen here is 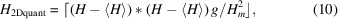
where 〈*H*〉 is the mean and *H*_*m*_ = max|*H*| is the maximum of the statistically evolving 1D sequence, and *g* is the maximum gray value magnitude.

In this work, the connection to HRMC is quite natural if the Fourier spectrum 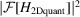 is viewed as a diffraction pattern, which ought to be constant across all spatial frequencies to ensure that *d*^*F*^ of Section 2.2.2 is optimized. We have thereby modified the HRMC algorithm to incorporate all of the metrics *M*^*f*^, *R*^0^, *f*^*z*^, *d*^*F*^*etc*. of Section 2.2.2, with the exception of κ due to the significant computational complexity of SVD. The adapted HRMC algorithm here minimizes a χ^2^ computed from the equation (10) array, relative to desired target values of all metrics.

A trial move of a randomly chosen radius power’s fractional sign *s*_*c*_ is accepted if χ^2^ goes down, otherwise a random number *r* on [0, 1] is chosen and compared with 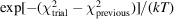 for conditional acceptance/rejection if *r* is smaller/larger, respectively, where *kT* is a global fictitious temperature. This differs slightly from conventional HRMC, whereby the temperature pertains only to the energy term. The purpose of the temperature here is to allow for simpler ‘simulated annealing’ optimizations, whereby all weighting factors can be gradually reduced (as done individually in conventional HRMC) to improve numerical efficiency. Similarly, the random step size of the randomly walked complex polynomial zero radii in equation (5) can be weighted by *kT* here to maintain an efficient accept/reject ratio for Monte Carlo trials. Further computational efficiency is achieved by gradually compressing the dynamic range *g* in equation (10) (similar to the iterative methods in Section 2.3.1).

**Figure 1:**
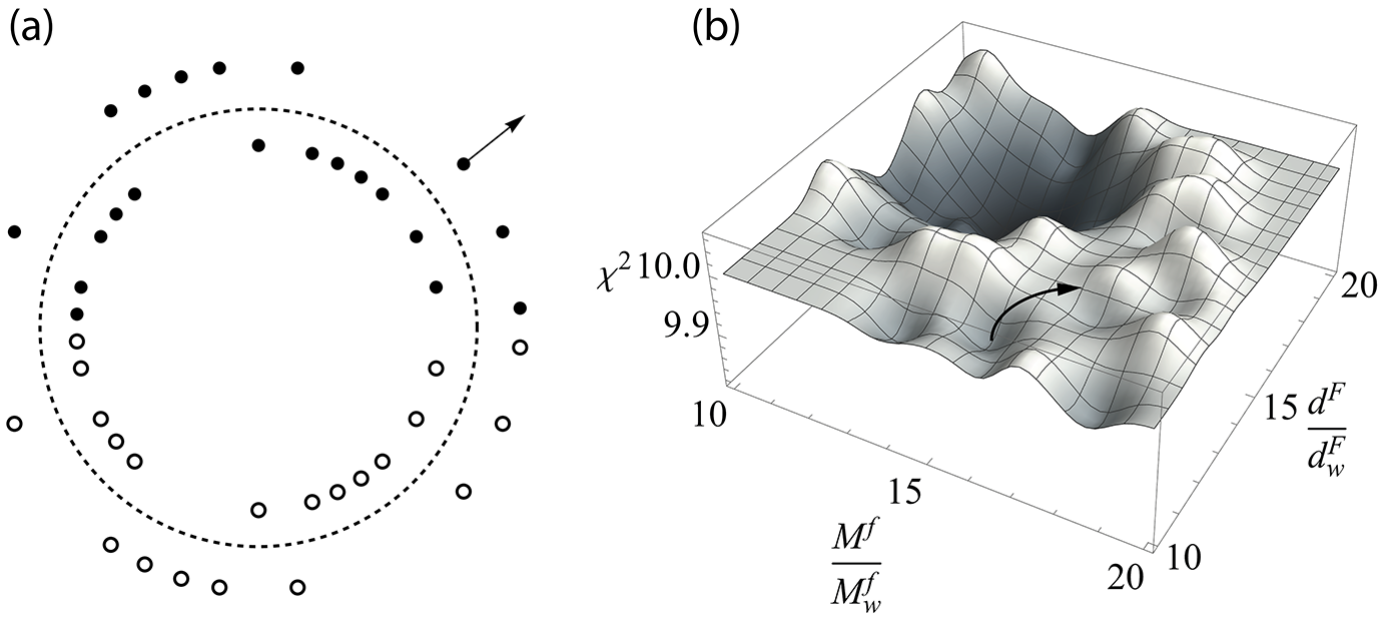
Hybrid reverse Monte Carlo of a 2D Huffman-like array, simplified to the case where only the merit factor *M*^*f*^ and spectral flatness *d*^*F*^ are to be optimized. A randomly chosen zero of the iteratively evolving 1D Huffman sequence defined by Huffman’s *P*(*z*) polynomial is moved in (*a*) by a small distance along a randomly chosen radial direction in the complex plane, as shown by the arrow. The corresponding conjugated zero (open circle vertically below in the bottom half plane) is accordingly moved in a mirrored fashion to ensure that the Huffman-like sequence remains real-valued. The curved arrow in (*b*) shows the change in the weighted 2D array autocorrelation metrics induced by the Markov event in (*a*), whereby the sum squared error χ^2^ slightly increases.

Fig. 1 ▸(*a*) shows an example Monte Carlo step, whereby the complex zeros of *P*(*z*) are initiated with a configuration of radius *R* or 1/*R*, pertaining to a canonical 1D Huffman sequence (the outer product of which yields a canonical 2D Huffman array).

Associated with the random trial move depicted in Fig. 1 ▸(*a*), Fig. 1 ▸(*b*) shows a possible increase in χ^2^, as a function of weighted *M*^*f*^ and *d*^*F*^ values, for a simplified HRMC simulation where only these autocorrelation metrics are to be optimized. The target values of *M*^*f*^ and *d*^*F*^ reside in the apparent deep global minimum in Fig. 1 ▸(*b*) of this example. For large fictitious temperature *kT*, such a small random increase in χ^2^ will likely be conditionally accepted, allowing the χ^2^ error to evolve in the space of the autocorrelation metrics, without becoming trapped in local minima. After sufficient sampling of this metric space, *kT* can be gradually reduced to efficiently optimize the quantized 2D Huffman-like array and ideally reach the global minimum. At the end of the annealing, the original *P*(*z*) zeros have randomly perturbed radii, different from the {*R*, 1/*R*} starting configuration, and the corresponding 1D real-valued sequence defining the 2D integer-valued Huffman-like array will no longer be canonical.

Further details appear in the supporting information, in addition to a simplified HRMC simulation example for creating a 2D Huffman-like array.

#### 2.3.3. Reformatting Huffman arrays as binary area-weighted masks

We forecast that the practical fabrication of masks with microscopic feature size and multiple uniform steps of transmitted X-ray intensity would be technically challenging. As a precautionary measure, we designed equivalent Huffman-like masks that had binary intensity transmission. These masks emulate multiple intensity levels by depositing absorbing material to cover fixed fractions of the pixel area.

For diffuse X-ray probes defined by Huffman-like array masks, one can sacrifice the spatial resolution of array elements in order to spread a given integer value across an effective ‘sub-pixel’ (or ‘sub-voxel’ for *n*D arrays), which we will refer to as a sub-element array. We have implemented an entirely binary approach here which preserves the auto-correlation behavior of the range-compressed and optimized Huffman-like arrays of Section 2.3.1 and Section 2.3.2.

For a given element of *P* or *N* in Section 2.2.1, the non-negative integer value can be decomposed as a binarized sub-element array *Se* comprising values either zero or unity, which we assume to be square for simplicity. We refer to a correlating/convolving sub-element array as *Se*_*c*_ and a given de-correlating array as *Se*_*d*_.

To preserve the cross-correlation of *H* = *P* − *N*, the inner product between any pair of sub-element arrays *Se*_*c*_, *Se*_*d*_ must match the products between pairs of elements of *H*. Denoting a sequence *b*_*s*_ of zeros and ones, a simple choice is the outer product *Se*_*c*_ = 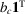 and *Se*_*d*_ = 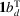, where 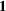 is a sequence of unit-valued elements (an identity array for generalization to binarized sub-voxels). In other words, *Se*_*d*_ is geometrically orthogonal to *Se*_*c*_, such that their inner product is simply the number of ones in *b*_*c*_ times that in *b*_*d*_. For arrays with transpose symmetry, the encoding and decorrelation scheme of equation (8) remains valid. A more general scheme that need not assume this symmetry is described in the supporting information.

The aperiodic auto-correlations *H* ⊗ *H* and (*B*_*p*_ − *B*_*n*_) ⊗ (*B*_*p*_ − *B*_*n*_) are exactly the same when the latter is sampled over element shifts, with sub-element shifts deemed a form of oversampling. To ensure precise integral sub-divisions, the binarized arrays *B*_*p*_ and *B*_*n*_ need be 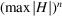 times larger than *H*, where *n* is the number of dimensions. For applications where the array size is a limiting factor, this increase emphasizes another reason why it is important to control the dynamic range of Huffman gray levels, 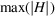.

When choosing how to subdivide binarized sub-element arrays, it is important to avoid correlated placements that could impair the delta-like performance of the entire Huffman array for sub-element shifts. As such, we have implemented random partitioning of any sub-element array into opaque and transmission sub-elements (*i.e.b*_1_, *b*_2_ generated randomly). However this randomized sub-structure (or many other binary placements) can be quite detailed, which implies impractical synthesis for physical manufacturing of entire *B*_*p*_ and *B*_*n*_ arrays. For the X-ray masks, we have hence implemented an alternative blocked-design, whereby sub-elements of equal value are grouped together as separate blocks of either zero or one, the binary ordering of which is randomly chosen within the sub-element array. Explicit numeric examples of 2D sub-element arrays and a binarized Huffman-like array are visualized in the supporting information.

## 3. X-ray scanning probe mask fabrication

Given that we now have methods to design masks as diffuse probes with delta-like autocorrelation, we would like to evaluate the concept experimentally with X-rays. We designed Huffman-like arrays to be constructed as physical X-ray attenuation masks with array sizes ranging from 11 × 11 to 86 × 86 and seven transmitted intensity values (*i.e.* integer values ranging from −3 to +3 where 3 indicates transmission as close to 100% as physically achievable). Since negative transmission cannot be achieved, the values in each array were separated into positive (*P*) and negative (*N*) masks (as described in Section 2.2.1) each with four equally spaced transmitted intensities.

The physical masks were fabricated on silica (SiO_2_) wafers. This was imprinted with multiple layers of tantalum (Ta) through a combination of lithography and etching techniques. Several instances of each mask design were fabricated with array element (or ‘pixel’) sizes in the mask patterns varying from 8 to 20 µm. The masks fabricated with four intensity levels will be referred to as *quaternary masks*. Binary versions of these masks were also designed (as described in Section 4.1) with each mask ‘pixel’ divided into 3 × 3 subpixels. The masks fabricated with two intensity levels will be referred to as *binary masks*.

**Figure 2:**
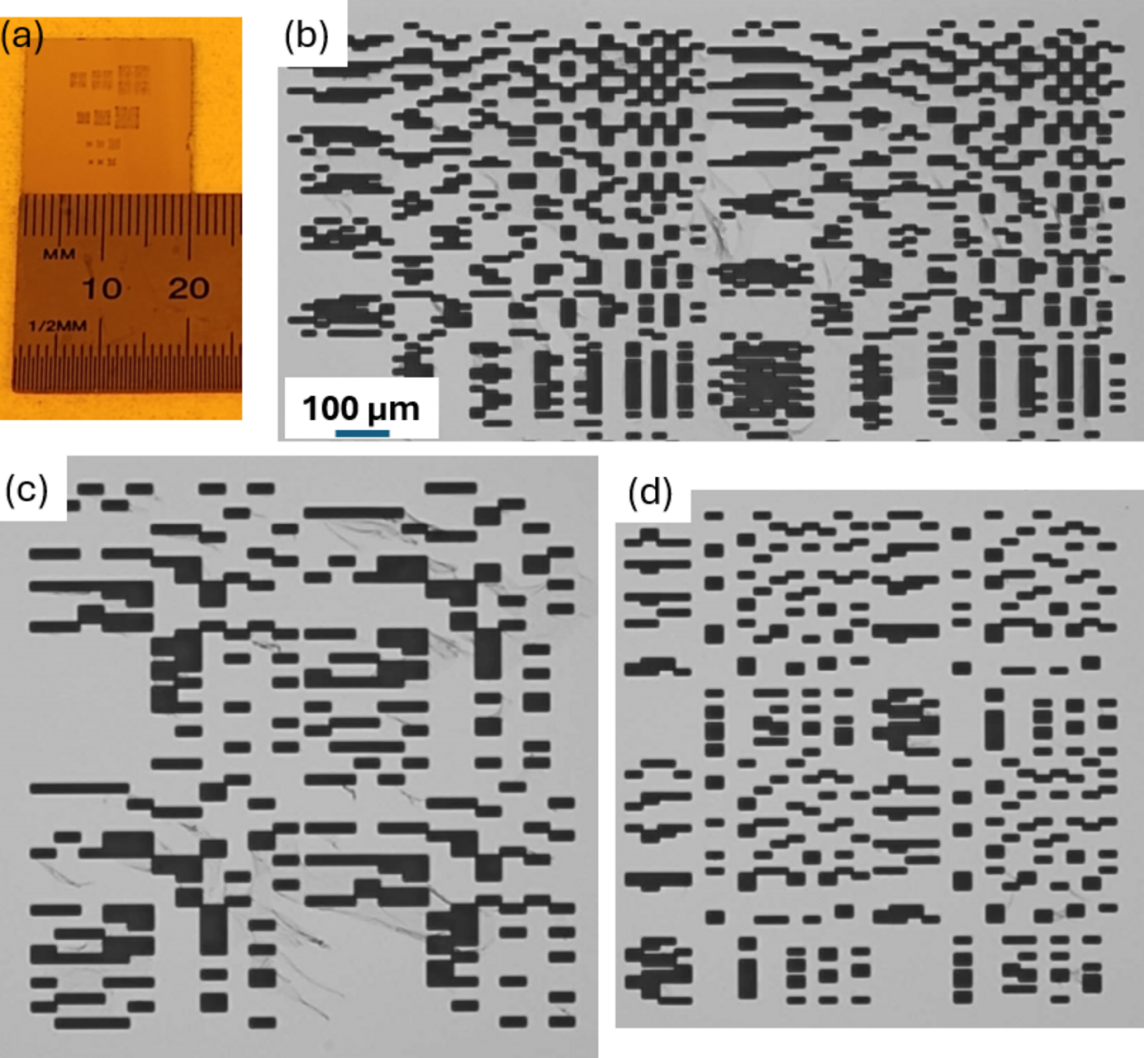
(*a*) One of the fabricated 2 cm × 2 cm substrates containing 12 binary Huffman-like masks. Optical images of (*b*) a *PN* pair of the fabricated 32 × 32 binary Huffman-like mask with 8 µm resolution, (*c*) a fabricated 11 × 11 binary Huffman-like mask with 15 µm resolution, and (*d*) a fabricated 15 × 15 binary Huffman-like mask with 10 µm resolution. The scale bar shown in (*b*) also applies for the images (*c*) and (*d*).

Binary masks were fabricated on 2 cm × 2 cm SiO_2_ wafers, which were coated with a 5 µm-thick Ta layer. 5 µm was, approximately, the maximum achievable film thickness using the sputtering technique, and Ta had relatively low X-ray transmissions (among the available materials) over the selected photon energies. More information is provided in the supporting information. An example fabricated wafer is depicted in Fig. 2 ▸(*a*). It includes 12 masks with four sizes (*i.e.* 11 × 11, 15 × 15, 32 × 32 and 43 × 43) and three different resolutions (*i.e.* 8 µm, 10 µm and 15 µm array subpixel sizes).

Optical images of the [*P*, *N*/*N*, *P*] masks for the 32 × 32 arrays with 8 µm pixel resolution, the 11 × 11 mask with 15 µm resolution and the 15 × 15 mask with 10 µm resolution are illustrated in Figs. 2 ▸(*b*)–2(*d*). Note that the streak artifacts in the optical images are residue from the photoresist; these streaks are near-transparent under X-rays and do not affect the functionality of the masks.

The fabrication process for the quaternary masks was more complex, although the same very large scale integration (VLSI) techniques were utilized. Quaternary Huffman-like masks require four levels {0, 1, 2, 3} with each level transmitting X-rays in steps of uniformly increasing intensity. Pixels at each level of the quaternary mask can be fabricated with a specific uniform thickness of Ta to provide the required level of X-ray transmission. Given the maximum achievable thickness of approximately 5 µm using the sputtering technique (as discussed in the supporting information), we can estimate the minimum X-ray transmission through our mask as approximately 17.5% at 12.4 keV; this is the transmission through level 0. 12.4 keV is just above the *L* absorption edges of Ta, providing maximum attenuation of Ta while still allowing transmission through silica. Requiring X-ray transmission, *T*, the thickness, *t*, of each level can be measured as *t* = −ln(*T*)/μ, where μ is the linear attenuation coefficient [0.3605 µm^−1^ for Ta at 12.4 keV (Berger *et al.*, 2010 ▸)]. The X-ray transmission and Ta thickness for each level is shown in Table 2 ▸.

**Figure 3:**
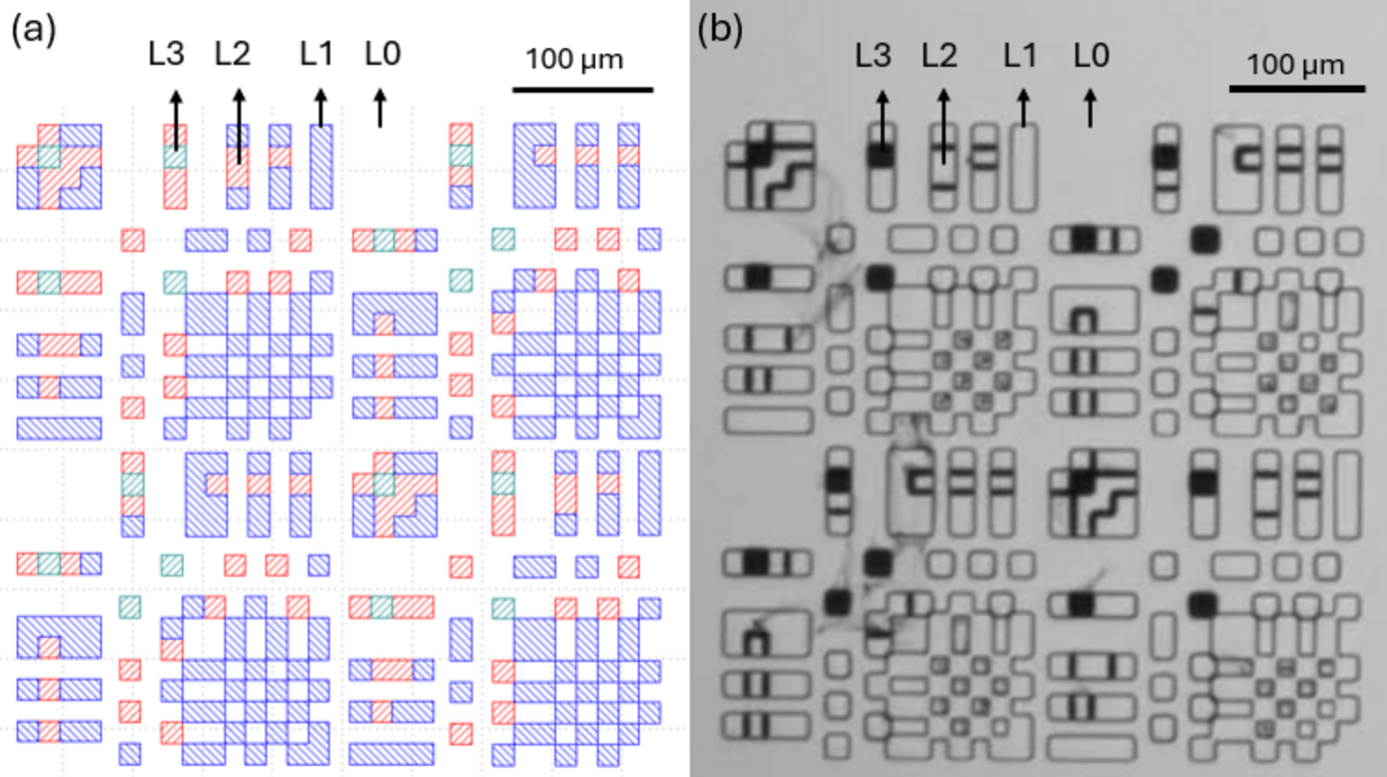
(*a*) Design and (*b*) optical image of the fabricated 15 × 15 quaternary Huffman-like mask. L0, L1, L2 and L3 represent level 0, 1, 2 and 3 of the mask, respectively.

Levels 1, 2 and 3 were fabricated through three lithography steps (one for each level) followed by three etching steps. Different etching times were required to achieve different thicknesses (see the supporting information for more detail). We fabricated 15 quaternary masks on a 4-inch SiO_2_ wafer. The masks were fabricated with five array sizes (11 × 11, 15 × 15, 32 × 32, 43 × 43 and 86 × 86) and three resolutions (10 µm, 15 µm and 20 µm pixel sizes). An optical image of the fabricated 15 × 15 quaternary mask is depicted in Fig. 3 ▸(*b*). The four Ta levels are indicated in the image. Note that level 0 (L0) is the substrate, which was coated with approximately 5 µm Ta.

## 4. Validation of mask fabrication

Before employing the masks in diffuse scanning probe experiments, the masks were examined at the Micro-Computed Tomography (MCT) beamline at the Australian Synchrotron. We validated the quality of the mask fabrication by imaging the masks using a 2D pixelated X-ray detector using 20 keV and 12.4 keV X-ray illumination. Based on the transmitted intensity images, we assessed the faithfulness of the mask structure and uniformity of transmission levels compared with the ideal array designs.

### 4.1. Binary masks

**Figure 4:**
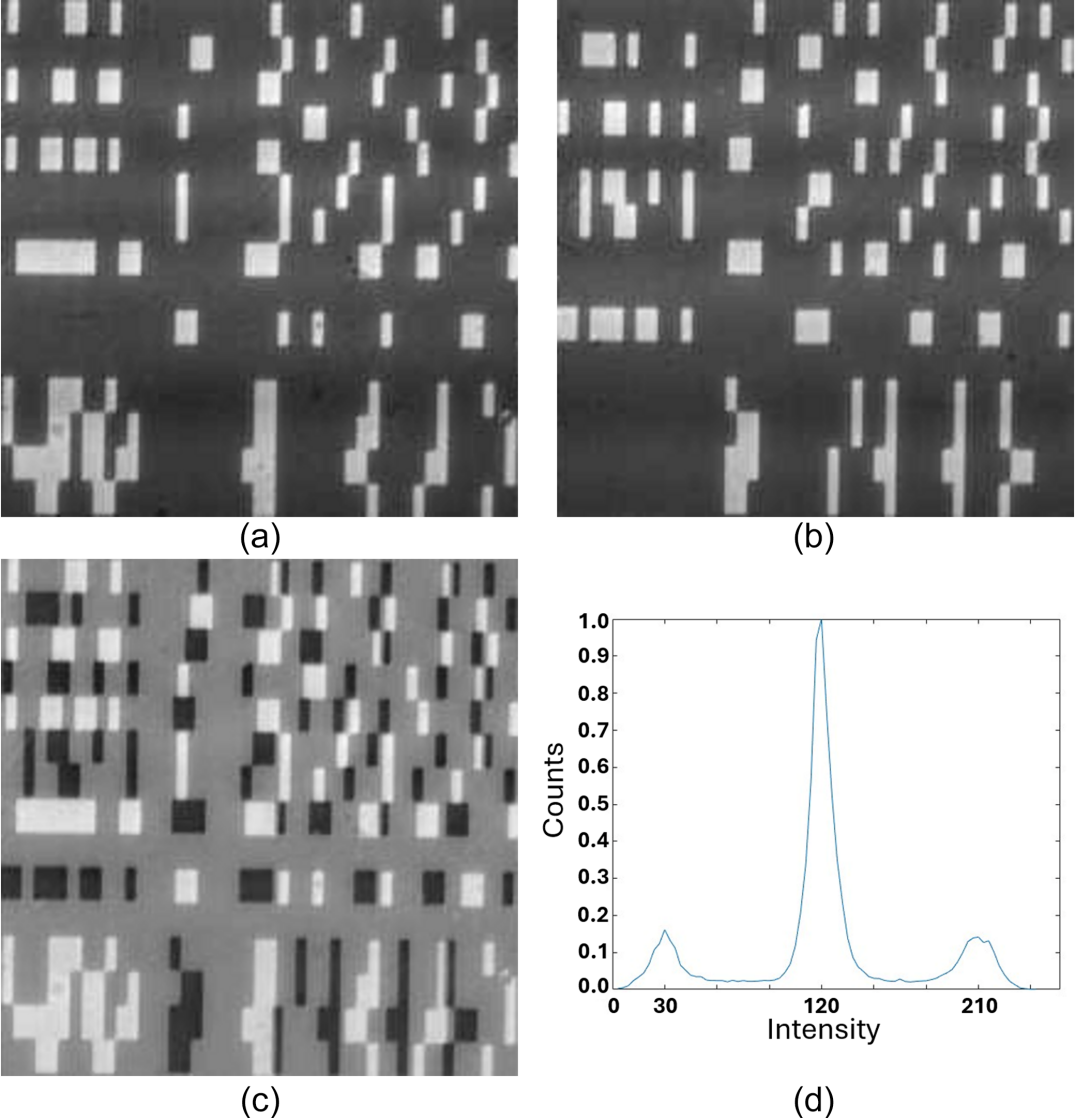
Detector images of the (*a*) positive (*P*) and (*b*) negative (*N*) 15 × 15 pixel Huffman-like mask generated using 3 × 3 binary pixels of pitch 15 µm. (*c*) The signed mask, re-generated as *P* − *N*. (*d*) Histogram of image (*c*). The three peaks show the distribution of the measured X-ray illumination intensities that passed through the −1, 0, +1 sections of the fabricated mask, respectively. The vertical axis in (*d*) is counts; the horizontal axis displays the relative signal intensity.

An example of the binary mask transmission pattern for the 15 × 15 pixel *P* and *N* regions with 15 µm subpixel pitch is shown in Figs. 4 ▸(*a*) and 4 ▸(*b*). The ‘reassembled’ Huffman-like array, calculated as *P* − *N*, is presented in Fig. 4 ▸(*c*). The histogram of measured X-ray intensities transmitted in this reassembled array is presented in Fig. 4 ▸(*d*). Here the X-ray beam energy was 20 keV with a 3% bandpass.

### 4.2. Quaternary masks

Images were acquired of the transmission of X-rays through the [*P*, *N*/*N*, *P*] mask arrangement for the 11 × 11 quaternary mask with 10 µm and 20 µm pixels, for the 15 × 15 quaternary mask with 10 µm and 20 µm pixels and for the 32 × 32 mask with 20 µm pixels. Here the X-ray beam energy was 12.4 keV with 1% bandpass to reduce variation in the energy-dependent transmission.

**Figure 5:**
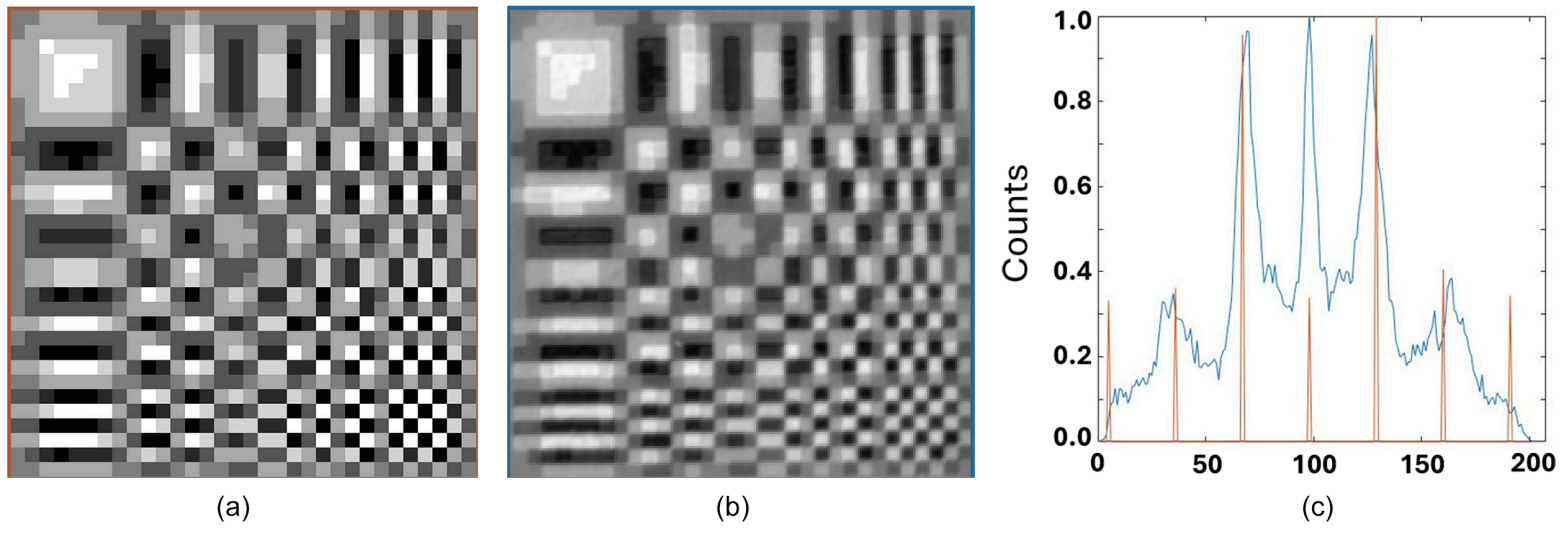
(*a*) Image of an ideal 32 × 32 ± 3 gray-level Huffman-like mask. (*b*) Image of X-ray transmission through the fabricated mask with 20 µm pixel size, presented as *P* − *N*. (*c*) Histogram of graylevels in the ideal mask, image (*a*) (shown in orange), registered with the histogram of (*b*) the intensity of the X-rays transmitted through the fabricated mask (shown in blue). Both image histograms show seven closely matched peaks, representing the relative X-ray transmission intensities for mask levels −3, −2, −1, 0, +1, +2, +3. The vertical scale is counts; the horizontal axis is relative intensity.

As an example, the 32 × 32 Huffman-like array is presented in Fig. 5 ▸(*a*). The reassembled array generated from the *P* and *N*X-ray images of the 32 × 32 quaternary mask with 20 µm pixel size is presented in Fig. 5 ▸(*b*) for comparison. The mask appears to produce a faithful representation of the ideal array. The histogram of this reassembled Huffman-like array is presented in Fig. 5 ▸(*c*). That histogram closely follows the spacing and relative intensities of the histogram of the ideal array, shown as the seven narrow peaks scaled and overlaid on the plot of measured X-ray intensities. The higher frequency observed around the central ‘zero’ intensity peak results from the entries generated along the edges of pixels where the measured images of the fabricated *P* and *N* meet and have their intensities subtracted to form the *P* − *N* image (hence creating extra zero pixels).

**Figure 6:**
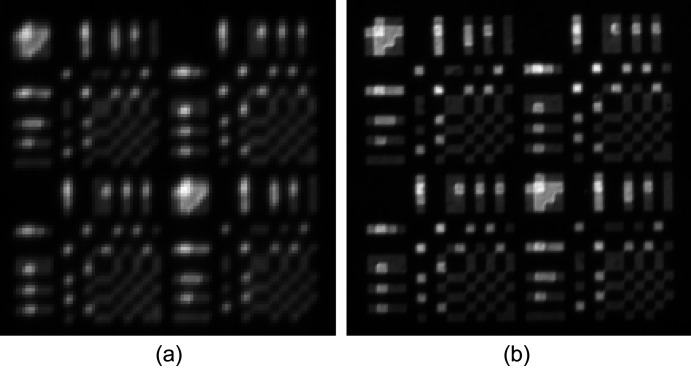
(*a*) Image of X-ray transmission through the [*P*, *N*/*N*, *P*] fabricated 15 × 15 gray-level masks with (*a*) 10 µm pixels and (*b*) 20 µm pixels.

**Figure 7:**
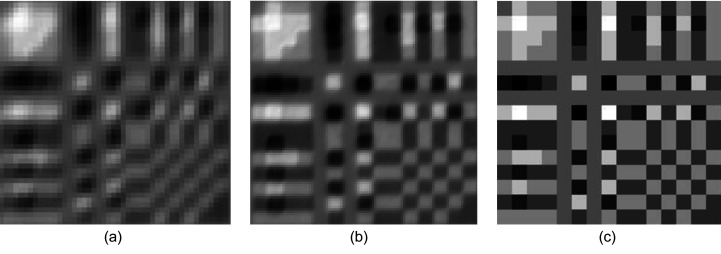
The 15 × 15 gray-level masks formed from Fig. 6 ▸ as *P* − *N* with (*a*) 10 µm pixels and (*b*) 20 µm pixels. (*c*) The ideal mask for comparison.

Fig. 6 ▸ shows two example ‘raw’ X-ray transmission images of the quaternary masks for a 15 × 15 array at two different pixel sizes (10 µm and 20 µm). The mask image data were acquired using a long exposure (or were summed over multiple repeated exposures) to reduce noise. The re­assembled Huffman-like arrays using these masks are presented in Figs. 7 ▸(*a*) and 7 ▸(*b*) to be compared with the ideal array in Fig. 7 ▸(*c*). Strong agreement between each original and fabricated mask is observed. Ta thickness here was changed by three successive depositions and etchings, resulting in some small variations around the pixel edges. These variations are much less evident for the 20 µm pixel fabrication than for the 10 µm pixel fabrication.

## 5. Experimental scanning probe method

### 5.1. Experiment set-up

The masks were employed to pattern diffuse scanning probes at the MCT beamline at the Australian Synchrotron. A monochromatic X-ray beam with energy of 20 keV was first used to analyze the performance of the binary masks. Note that the binary masks can be used across a range of photon energies, since they contain only opaque and transparent parts. In contrast, the quaternary masks require each level to transmit a specific percentage of the incident beam. The thicknesses of Ta to give each transmission fraction were optimized for a single photon energy (12.4 keV in this case).

**Figure 8:**
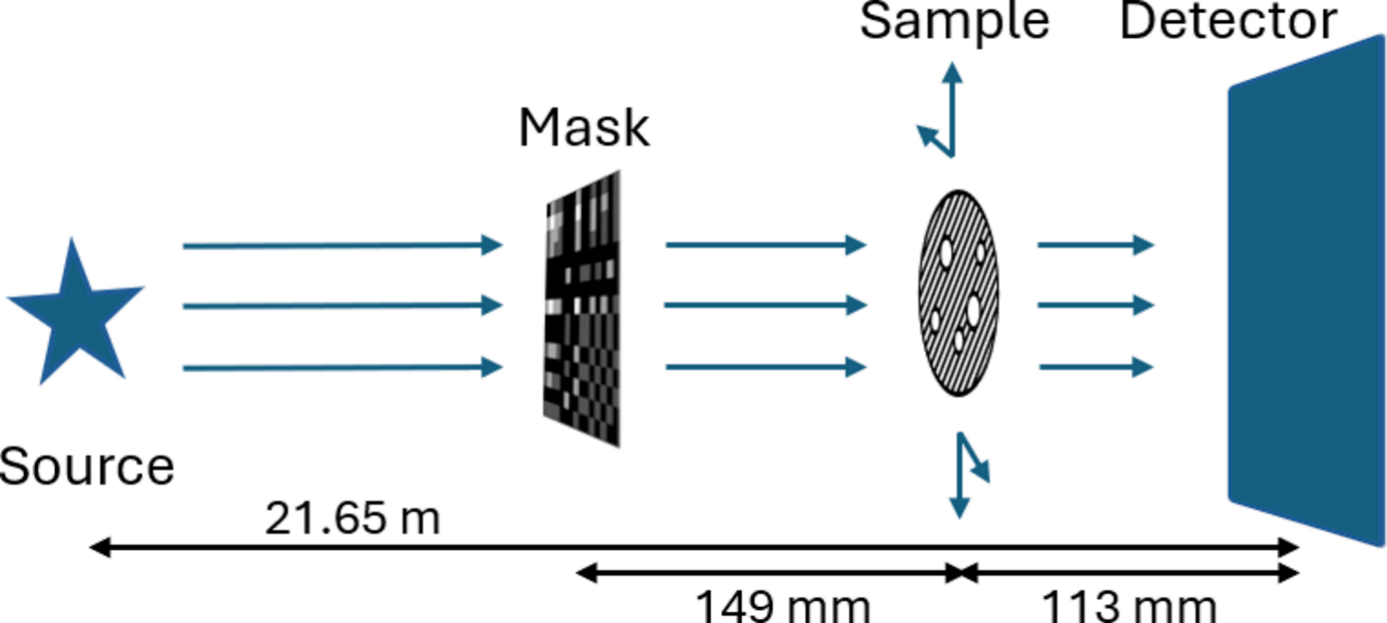
Experimental geometry on the MCT beamline at the Australian Synchrotron. The mask, sample translation and detector planes define the coordinates (*x*, *y*), while the beam propagates in the +*z* direction.

A schematic of the experimental setup is shown in Fig. 8 ▸. The mask was placed approximately 262 mm from the detector, and the sample was located approximately 149 mm in front of the mask. The sample was mounted on a moving stage to allow the test object to be transversely scanned over the selected mask. A 2D pixelated detector was utilized throughout the entire process. The resulting images were then processed digitally into four individual sensors (or buckets), one for each quadrant of the [*P*, *N*/*N*, *P*] arrays. As each scan proceeded, these bucket values were collected into bucket arrays for image reconstruction, as described in Section 5.3.

### 5.2. Test objects

The performance of the Huffman-like probes was examined with a range of test objects specifically designed and fabricated for this purpose. We started with simple pinholes and progressed to more complex objects. Scanning a pinhole test object is of interest as the resulting bucket image should reproduce an exact image of the discretely shaped X-ray beam intensity. This image is obtained with very weak illumination, as the entire bucket receives information from just one mask pixel at each scan point. The pinhole imaging results test the resilience of broad Huffman-like array imaging under very low signal-to-noise conditions.

**Figure 9:**
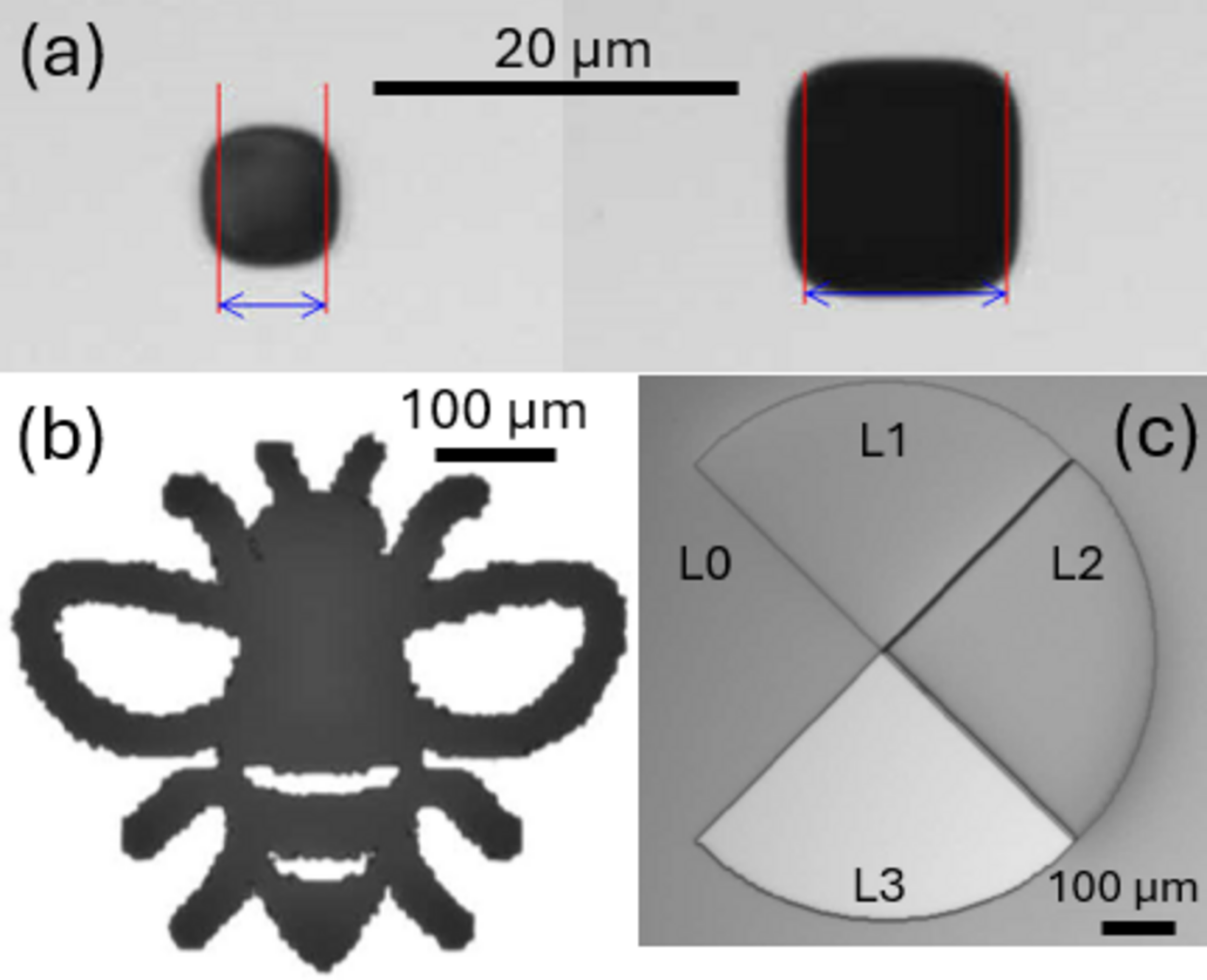
Optical images of some of the fabricated test objects: (*a*) 5 µm and 10 µm pinholes, (*b*) a binary object (bee image) and (*c*) disk with four quadrants, each quadrant having a different uniform thickness (L3 < L2 < L1 < L0).

Four pinholes, with diameters of 5 µm, 10 µm, 15 µm and 20 µm, were fabricated to be imaged using different mask sizes. Optical images of two fabricated pinholes are illustrated in Fig. 9 ▸(*a*). The material and fabrication process were the same as those used for the binary Huffman-like mask explained in Section 3. Utilizing the same procedure, multiple binary objects of different sizes were also fabricated. An optical image of a binary test object is shown in Fig. 9 ▸(*b*). We also fabricated gray-level objects using the four-level fabrication method described in Section 3. Fig. 9 ▸(*c*) shows an example of a gray-level test object, where each quadrant of the circle has a unique, uniform thickness of Ta.

### 5.3. Experimental procedure

The object needs to be over-scanned on each edge by at least the full size of the mask to obtain valid bucket signals able to accurately deconvolve up to the object edges. Thus the acquisition time for 2D images and the size of the required bucket data scales with the object size, desired spatial resolution and the chosen array size of the mask.

We first scanned different pinholes (one at a time) over a selection of binary masks. As described above, the number of images collected for each scan depended on the array size of the masks. For instance, 31 × 31 images were collected for a 15 × 15 array mask to cover the entire [*P*, *N*/*N*, *P*] mask. The exposure time was 0.03 s for each position in these scans. In addition to the scans, we collected ten flat-field (FF) and ten dark-field (DF) images, as well as ten mask-field (MF) images (*i.e.* images of the mask only). Utilizing the binary masks, we also scanned other test objects including a bee and a gray-level circle, as shown in Figs. 9 ▸(*b*) and 9 ▸(*c*), respectively.

After scanning multiple objects with binary masks using 20 keV X-rays, we changed the photon energy to 12.4 keV and repeated similar experiments using the quaternary masks. The exposure time was increased to 0.2 s to improve the signal-to-noise ratio. We first scanned a pinhole over several 11 × 11 quaternary masks and then progressed to 15 × 15 and 32 × 32 masks. In addition to the pinholes, a few binary and gray-level objects were scanned using 11 × 11 and 15 × 15 quaternary masks. Again, ten images of the FF, DF and MF were also collected for each scan.

**Figure 10:**
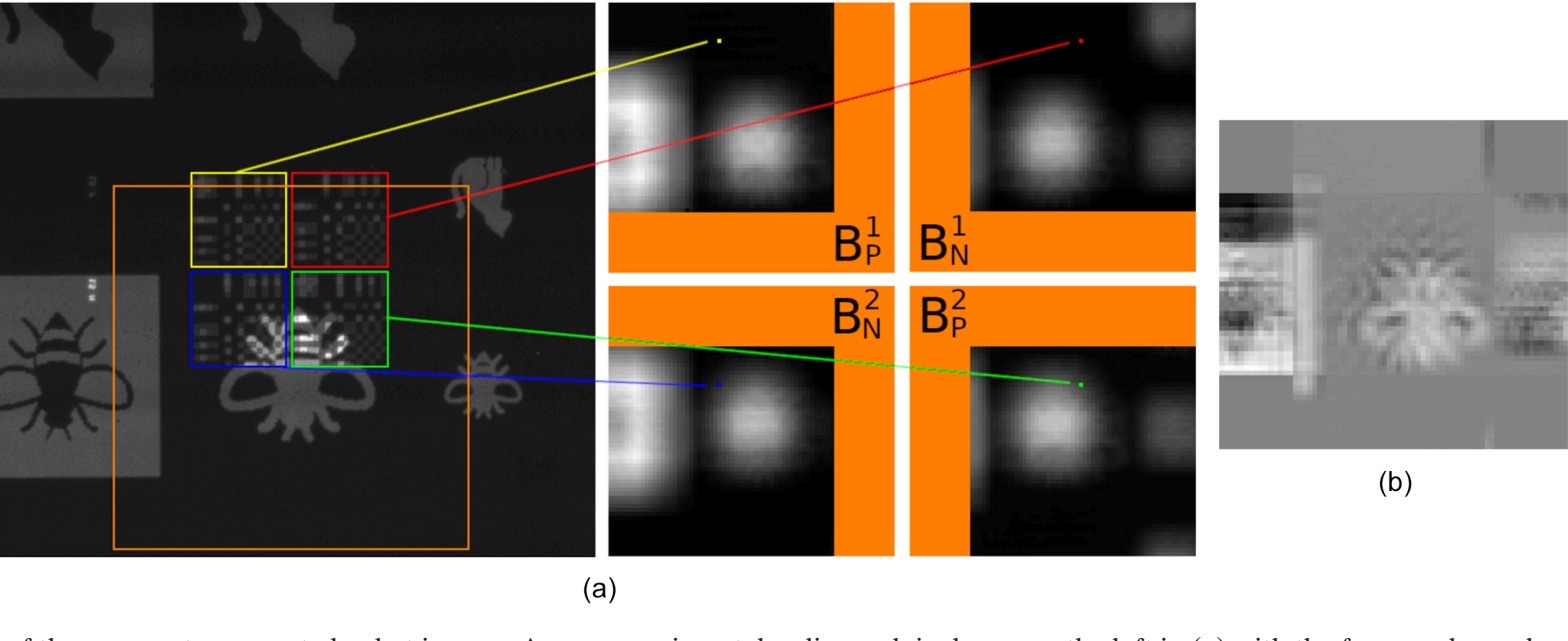
An example of the process to generate bucket images. A raw experimental radiograph is shown on the left in (*a*) with the four mask quadrants identified using colored squares. The center of the mask region is scanned around in the orange square. At each position integrated pixel values in each quadrant are added to the respective pixels of the bucket images, 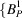, 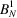, 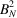, 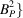, for each quadrant on the right. Note the *P* and *N* masks individually produce bucket values that form a low-pass filtered image of the object being scanned. The positive, 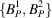, and negative, 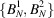, bucket image pairs are combined and subtracted to form the diffuse-probe scanned image shown in (*b*). This image is deconvolved to produce the result in Fig. 13 ▸(*a*-iii).

Once the sets of 2D pixelated images were collected for each scan, a series of postprocessing analysis was performed to create four bucket images for each scan:

(i) Average the ten FF and ten DF images.

(ii) Subtract the averaged DF image from both the FF and scanned images.

(iii) Normalize the scanned images by dividing by the FF image.

(iv) Identify the coordinates of each mask quadrant [see colored squares in the example radiograph in Fig. 10 ▸(*a*)].

(v) Integrate the measured intensities over each quadrant into separate bucket images *B*_*N*_ and *B*_*P*_ [depicted for an example raw experimental radiograph in Fig. 10 ▸(*a*)].

(vi) Combine the aligned and normalized *P* and *N* bucket images as *B*_*P*_ − *B*_*N*_ to give the result of scanning the object with a Huffman-like array [depicted for an example in Fig. 10 ▸(*b*)].

(vii) Deconvolve the combined bucket image using the ideal Huffman-like array to reconstruct an image of the scanned sample.

## 6. Experimental results: reconstructed X-ray images

Here we present the results obtained using diffuse X-ray scanning probes shaped by our masks that are based on Huffman-like arrays. The test object images are reconstructed from the scanned data (or *bucket signals*) obtained using both binary and quaternary fabricated masks of different sizes and spatial resolutions. The scans are performed by raster scanning the objects across the stationary patterned illumination probe. Results for the 15 × 15 binary mask and 32 × 32 quaternary mask are shown in the following sections. In both cases, the first object is a pinhole aperture with dimensions similar to that of the mask pixels. The pinhole test also demonstrates the resolution of the process. Following that, simple binary objects were scanned, and finally multilevel (gray) objects were imaged. Note that there is no difference in computing the reconstructed images, for each case.

### 6.1. Pinhole images: binary mask

**Figure 11:**
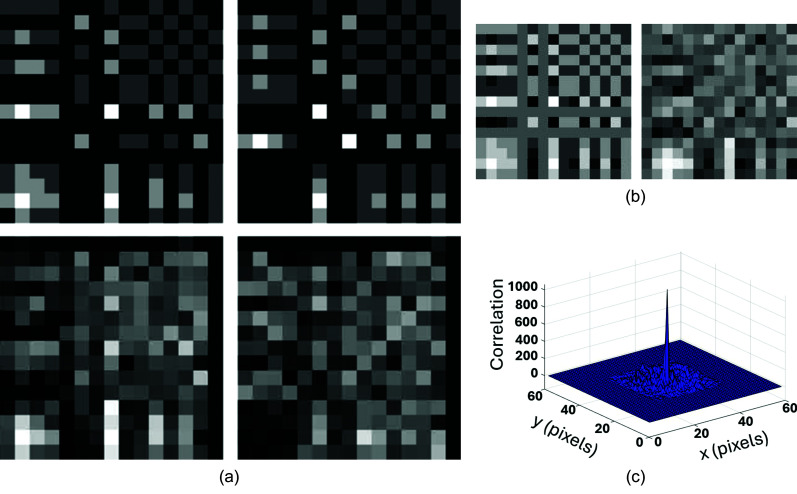
Pinhole images of the binary 15 × 15 mask. (*a*) Top row: original *P* and *N* integer arrays, black = 0, white = 3. Bottom row: X-ray bucket images obtained for the *P* and *N* mask. (*b*) Left: original Huffman-like array, black = −3, white = +3. Right: reconstructed X-ray pinhole image of the 15 × 15 Huffman-like mask. (*c*) A surface plot of the cross-correlation of the original integer array [left image of (*b*)] with the reconstructed X-ray-image of the mask [right image of (*b*)]. The correlation is delta-like. The vertical scale has been normalized to set the maximum of the central peak to have value 1000.

The 15 × 15 binary mask, in [*P*, *N*/*N*, *P*] form, has *P* and *N* masks with 45 × 45 subpixels (due to the 3 × 3 subpixel area-weighting used to accommodate the gray levels [0, 1, 2, 3]). The binary mask, fabricated with 8 µm subpixels, was used to illuminate a 20 µm pinhole. This pinhole almost covers the physical 24 µm width of the full-size mask pixel, *i.e.* 3 × 3 subpixels. The pinhole was 2D raster scanned, using 24 µm steps, to produce a 15 × 15 bucket image. Fig. 11 ▸(*a*) shows the ideal *P* and *N* integer arrays and, below, the mean of the two *P* and two *N*X-ray bucket images. On the left of Fig. 11 ▸(*b*) is the ideal Huffman-like ±3 valued array for comparison with the reassembled X-ray bucket image, *i.e.P* − *N*, shown on the right. The X-ray pinhole mask image clearly resembles the original Huffman-like array. The surface plot in Fig. 11 ▸(*c*) shows the reassembled Huffman-like image cross-correlated with the ideal array.

The binary masks with 8 µm subpixels were fabricated with an 8 µm gap separating the *P* and *N* boundaries of the [*P*, *N*/*N*, *P*] layout. The 24 µm steps used to scan the pinhole were thus spatially misaligned by a third of a pixel after stepping across the *P* and *N* boundary gaps. The raster scan axis was also rotated by about a degree relative to the mask axis, meaning the scan and mask locations had some shear. The end-points of the raster scan were terminated nearly a pixel short of the mask boundaries, as evident in Fig. 11 ▸. Despite the imaging misalignment and the low signal-to-noise ratio, the correlation of this diffuse Huffman-like scanned image with the original array proved to be delta-like, reconstructing the pinhole faithfully [see Fig. 11 ▸(*c*)].

### 6.2. Pinhole images: quaternary mask

**Figure 12:**
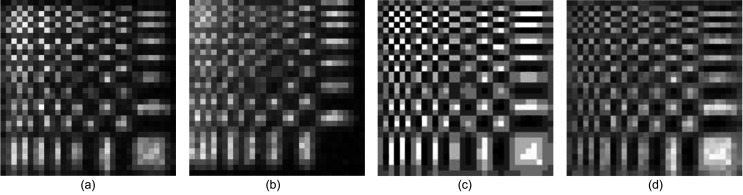
Pinhole images of the 32 × 32 quaternary mask. (*a*) Mean X-ray bucket image obtained for the *P* mask. (*b*) Mean bucket image for the *N* mask. (*c*) Original Huffman-like array, black = −3, white = +3. (*d*) Reconstructed X-ray pinhole image of the 32 × 32 Huffman-like mask.

The 32 × 32 quaternary mask was fabricated with 20 µm subpixels. This mask was used to illuminate a 20 µm pinhole with a beam of 12.4 keV X-rays. The pinhole was 2D raster scanned in 20 µm steps to produce 32 × 32 *P* and *N* bucket images. The mean of the two resulting *P* and mean of the two *N*X-ray bucket image measurements are shown in Figs. 12 ▸(*a*) and 12(*b*), respectively. These bucket images were re­assembled, as *P* − *N*, to recover the Huffman-like scanning probe image, as shown in Fig. 12 ▸(*d*). The X-ray Huffman-like probe image very closely resembles the ideal Huffman-like array depicted in Fig. 12 ▸(*c*).

The cross-correlation of the 32 × 32 Huffman-like X-ray probe image with the ideal array was delta-like, thereby reconstructing the pinhole well. The values in the area around the peak are given in Table 3 ▸. This result confirms that the broad X-ray probe (with a footprint spread over an area of 1024 pixels) is indeed able to be sharply focused to a pixel-like point when deconvolved.

### 6.3. Binary and gray-level object images: binary mask

**Figure 13:**
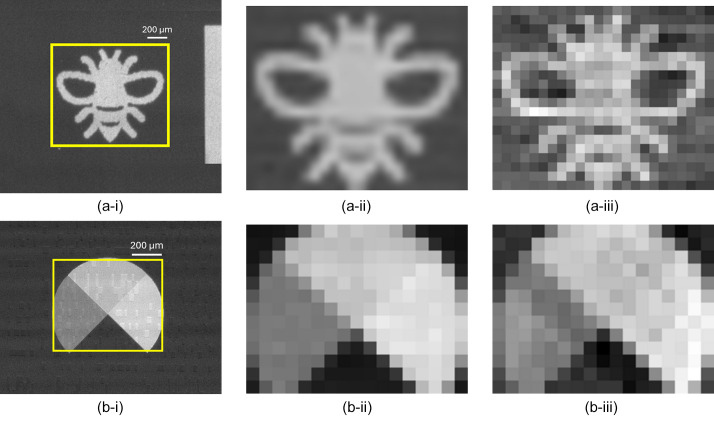
(*a*-i) Binary image of a bee, and (*b*-i) multiple gray-level circle with quadrants of different intensities. (iii) Recovered images compared with (ii) the expected images using a quaternary 15 × 15 Huffman like array created using 3 × 3 binary subpixels with pitch 15 µm.

Fig. 13 ▸(*a*-i) shows, in the highlighted region, an X-ray radiograph of a binary object (a ‘bee’) to give context of the surrounding region that is also included to some degree in the scan. Fig. 13 ▸(*a*-ii) shows this region rescaled and binned to the same FOV and pixel size as the reconstructed image in Fig. 13 ▸(*a*-iii). This last image is the Huffman-like X-ray probe image obtained from the 15 × 15 binary mask (with 15 µm pixels) scanned over this object, after being deconvolved by the ideal Huffman-like array. The radiograph in Fig. 13 ▸(*a*-ii) and reconstructed diffuse scanning-probe image in Fig. 13 ▸(*a*-iii) show close agreement.

Fig. 13 ▸(*b*-i) shows an X-ray radiograph of a circular disk, printed with subquadrants of different but uniform Ta thickness to provide a (gray-level) range of transmitted intensities. Fig. 13 ▸(*b*-ii) shows this region rescaled and binned to the same FOV and pixel size as the reconstructed image in Fig. 13 ▸(*b*-iii). Fig. 13 ▸(*b*-iii) was obtained after deconvolving the *P* − *N* Huffman-like X-ray probe image obtained by scanning the 15 × 15 binary mask (with 15 µm subpixels) over this ‘gray’ disk. As for the binary mask results, the radiograph in Fig. 13 ▸(*b*-ii) and the reconstructed diffuse scanning-probe image in Fig. 13 ▸(*b*-iii) show close agreement.

### 6.4. Binary and gray-level object images: quaternary mask

**Figure 14:**
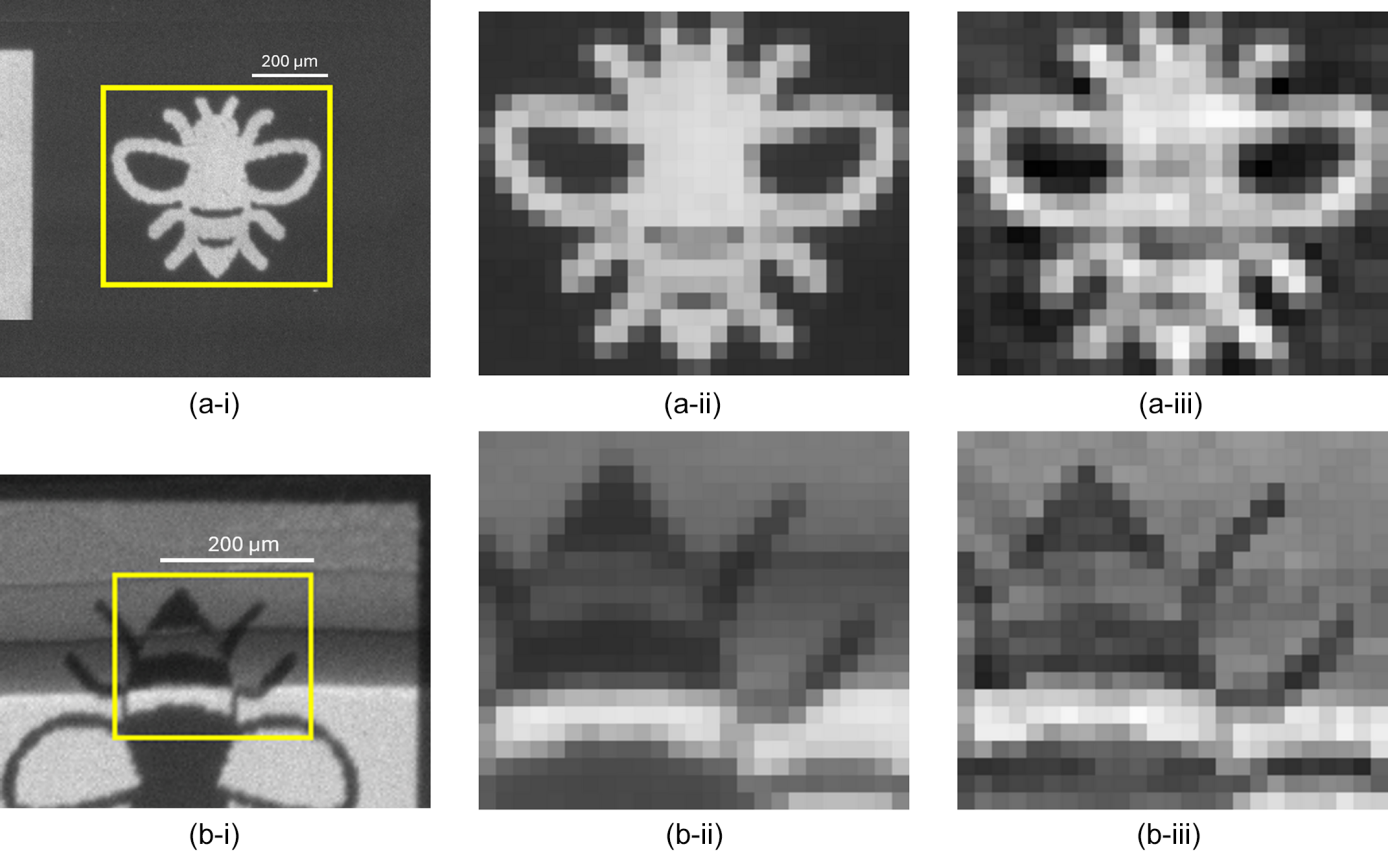
(*a*-i) Image of a binary bee and (*b*-i) a multiple gray-level bee partially covered with several folded layers of Al tape. The recovered images (*a*-iii) and (*b*-iii) compare well relative to the expected images (*a*-ii) and (*b*-ii), respectively. The 15 × 15 Huffman-like arrays were created from quaternary masks using pixels with pitch: (*a*) 10 µm, (*b*) 20 µm.

Fig. 14 ▸(*a*-i) shows, in the highlighted region, a projected X-ray image of a binary object (a ‘bee’). Fig. 14 ▸(*a*-ii) shows this region rescaled and binned to the same FOV and pixel size as the reconstructed image in Fig. 14 ▸(*a*-iii). The image in Fig. 14 ▸(*a*-iii) shows the *P* − *N* Huffman-like X-ray probe image obtained from the 15 × 15 mask (with 10 µm pixels) scanned over this object, after being deconvolved by the ideal Huffman-like array.

Fig. 14 ▸(*b*-i) shows an X-ray radiograph of another printed (‘bee’) object, this time combined with several layers of aluminium folded into strips across the bee body to provide a range of object thicknesses. Fig. 14 ▸(*b*-ii) shows the highlighted region rescaled and binned to the same FOV and pixel size as the reconstructed image in Fig. 14 ▸(*b*-iii). The image in Fig. 14 ▸(*b*-iii) was obtained from the *P* − *N* Huffman-like X-ray probe image obtained by scanning the 15 × 15 mask (with 20 µm pixels) over this ‘gray’ object. The radiographs in Figs. 14 ▸(*a*-ii) and 14 ▸(*b*-ii) compare well with the Huffman-like mask reconstructed images in Figs. 14 ▸(*a*-iii) and 14 ▸(*b*-iii).

## 7. Discussion and future work

The patterns used here for diffuse X-ray scanning probes based on Huffman-like arrays have been shown to have the ability to produce high-resolution images. In this case the resolution is dictated by the scanning step size and mask pixel size rather than the overall probe beam dimensions. The tailored size and shape of these beams serve as a means to distribute the equivalent energy of a sharp probe over a much wider footprint. A broad, diffuse incident beam lessens the potential radiation damage to the specimen and reduces thermal/structural changes.

We note that larger masks can spread the same energy of an equivalent beam much more thinly. However, they are harder to design while also retaining a small range of gray levels and require more complicated structures. Also, masks with a large footprint require objects to be overscanned by at least the mask width outside the object edges to accurately recover internal detail out to the object edges. We observed that fabrication of masks with pixels of larger size (here 20 µm pixels rather than 10 µm) was shown to provide more uniform levels of transmission with more regular and sharply defined pixel edges.

Huffman-like masks, as differential filters, are signed operators. Masks that project positive beam intensities need to be split into positive, *P*, and negative, *N*, masks. The *P* and *N* masks are scanned individually over test objects, with *P* and *N* bucket values being collected individually (or as a group comprising several mask regions, *e.g.* [*P*, *N*/*N*, *P*]). This duplication of scanning in both row and column directions increases the total imaging time, including the need to over-scan the areas around the object being imaged. However, scanning with the [*P*, *N*/*N*, *P*] group of low-pass mask filters provides redundancy and choice in how to compute the differential Huffman-like mask, *P* − *N*, which can be composed in nine distinct ways [*i.e.* top-row differences *P*_*t*_ − *N*_*t*_, bottom row *P*_*b*_ − *N*_*b*_ or combinations (*P*_*t*_ + *P*_*b*_) − *N*_*t*_, *P*_*t*_ − (*N*_*t*_ + *N*_*b*_), *etc*.]. The difference in timing where each of the four masks synchronize to scan the same point of the object also permits the possibility of correcting for variations in the brightness and uniformity of the incident illumination.

Huffman-like masks with multiple transmission levels are shown here to perform better as imaging probes when used for the aperiodic scanning of objects than do binary masks based on perfect periodic arrays. Fabrication of masks with a smaller number of transmission levels is technically more robust, as significantly fewer sequential deposition and removal operations are required. However, fabricating Huffman-like arrays with a larger number of transmission levels would have permitted significantly better autocorrelation metrics. For example, allowing the integer range of our 11 × 11 Huffman-like arrays to increase to ±4 from ±3 would have improved the autocorrelation peak-to-side-lobe ratio to 26 from 20, the merit factor to 37 from 24, have flattened the Fourier spectra (0.47 from 0.52), and lowered the condition number (to 1.24 from 1.33).

Binary masks are simpler to fabricate (with single Ta layer deposition) but require spatially larger pixels since they must be divided into subpixels over which the area of ‘open’ transmission can be varied to accommodate a range of gray-level transmission of X-rays. The pixel area scales up with the range of transmitted intensities, further decreasing the spatial resolution of the probe. Orthogonal patterns of area filling of these sub-pixels are needed to reduce cross-pixel correlations. Any low-pass correlations between sub-pixels act to reduce the delta-like property of the masks.

An alternative method to make gray-weighted *P* and *N* masks that use a binary layer for transmission would be to switch pixel-sized micro-mirrors in or out for fractions of the total exposure time at each scan point. Such a method seems impractical for current X-ray imaging. However the Huffman-like masks developed here for X-rays are well suited for optical imaging applications. A recently published special issue (Dainese *et al.*, 2024 ▸) addresses the shaping of optical beams at sub-wavelength scales to achieve a wide variety of probe objectives.

We note that a major constraint on the experimental work reported here was the time required between acquiring successive raster-scan data points. A combination of beam switching, translation stage movement and detector resetting meant about 3 s were required for each scan point. This limitation prohibited us from scanning larger test objects. Even for a pinhole, we needed to over-scan the test objects by at least the width of the Huffman-like mask. Scanning a pinhole with a 32 × 32 mask in [*P*, *N*/*N*, *P*] mode then took over an hour to complete; the small bee images took longer. Future experiments should reduce the scan-time by several orders-of-magnitude, using steps that match the actual beam exposure time used per point.

In future experiments it may be useful to track the (*x*, *y*) co-ordinates of the center of the probe pattern as the beam propagates along the *z* direction. Similar work using Airy beams was reported by Zhou *et al.* (2020 ▸). The masks designed by Svalbe *et al.* (2021 ▸) may be useful here, as the delta-like mask planes also project as sharp delta-functions for several directions.

The design of 3D Huffman-like masks with a small range of intensity levels (±3) has been investigated briefly, with encouraging results for the construction of 11 × 11 × 11 voxel arrays that have good autocorrelation metrics and condition number (an example is given in the supporting information).

This diffuse-probe concept can be applied to existing scanning probe techniques such as X-ray fluorescence (XRF) imaging (Paunesku *et al.*, 2006 ▸). Some consideration of how to employ the *P* and *N* masks separately is required. XRF microscopy involves a microfocused (pencil beam) X-ray probe scanned over a small (100 µm to 10 mm diameter) object and the X-rays fluorescing from the object are recorded with an energy-dispersive detector. The samples are typically biological and the focused beam can damage the sample. A Huffman-like diffuse probe would minimize radiation damage at the cost of a slightly larger scanning range. Since beam masking is used rather than beam focusing, the concept could also enable such a technique to be applied more easily with a laboratory X-ray source. How this concept could translate to more complicated scanning techniques such as ptychography (Pfeiffer, 2018 ▸) is scope for future research. There are also future avenues to apply larger (high throughput) [*P*, *N*/*N*, *P*] Huffman array masks in a static configuration as effective lenses in an aforementioned coded aperture context (Fenimore & Cannon, 1978 ▸; Cieślak *et al.*, 2016 ▸), to enable low-dose pinhole images via large flux, with sharp reconstruction. A comprehensive study on spatial resolution and exposure dose is also within the scope of future work. To this end, the pinhole results of Section 6.1 and Section 6.2 demonstrate the requisite stability to mask imperfections and slight misalignment issues.

Such coded aperture decorrelation would require a pixel­ated camera, rather than the bucket detector used in this work. Pixelated cameras in conjunction with Huffman encoding could potentially enhance scanning image modalities that utilize cross-correlation, since 2D Huffman arrays possess sharp delta-like autocorrelation. One possible example includes the aforementioned strain-mapping in convergent beam electron diffraction, whereby a patterned 2D illumination aperture is tracked in raster-scanned far-field diffraction patterns using cross-correlation between the known binary mask and the aperture shape imprinted onto Bragg disks (Zeltmann *et al.*, 2020 ▸). In that setting, cross-correlation is used to sensitively map the expansion and contraction of Bragg angles due to spatial variations in strain, measured via robust detection of noisy Bragg disks. The binary forms of our [*P*, *N*/*N*, *P*] masks could be used in that setting as probe-forming apertures, to enhance the cross-correlation response via Huffman decoding of the far-field patterns. Given the small scale of electron microscope apertures and the complexity of our 2D binary designs, it remains to be seen whether lithographs of sufficient fidelity can be realized in this proposed implementation.

Another related potential application of Huffman encoding is for differential phase contrast imaging, which conventionally measures the refraction-induced deflection of a beam in the far-field (Dekkers & de Lang, 1974 ▸) or in the near-field (McCartney *et al.*, 1996 ▸; de Jonge *et al.*, 2008 ▸; Morgan *et al.*, 2011 ▸). For smoothly varying refraction, the sought deflections can be obfuscated by other sources of contrast that dominate deflection measurements such as the center of mass. To counter these issues in the context of spatially mapping magnetic refraction, it has been demonstrated that cross-correlation of circular aperture edges can separate the sought beam displacement from confounding artifacts such as strong modulation of Bragg diffraction contrast (Krajnak *et al.*, 2016 ▸). Again, the delta-like autocorrelation response of Huffman arrays appears ideally suited to enhance the tracking of refraction-induced deflections. Though not explored in this work, the Huffman mask could be transmitted through a refracting specimen using a raster scan much simpler than our encoding strategy, with deflections measured by pixelated images of displaced [*P*, *N*/*N*, *P*] masks in the near field. Similarly to the case of strain-mapping, one could also envisage measurements of this kind in the far-field, with the Huffman mask acting as a probe-forming aperture, for sufficiently small convergence angles in the illumination.

## 8. Summary and conclusions

We have successfully designed and fabricated a variety of Huffman-like masks, made as layers of tantalum deposited on a silica wafer. The masks, in sizes from 11 × 11 to 86 × 86 pixels, were fabricated with pixel widths that ranged from 8 µm to 20 µm. The masks were built to transmit discrete Huffman-like patterns of X-ray illumination with four distinct stepped levels of intensity.

The masks were exposed to near-monochromatic and uniform intensity incident X-ray beams. The radiographs of these masks, taken on a finely pixelated detector with 6.5 µm pixel pitch, bore close resemblance to the designed patterns. The autocorrelation of each reassembled mask X-ray image demonstrated a sharp delta-like pattern.

We acquired bucket signal images of various simple test objects using these masks. The bucket images from scanning the masks over a 20 µm pinhole aperture faithfully reproduced the designed array pattern. The deconvolved bucket X-ray images of the pinhole apertures also produced a delta-like correlation that confirmed that the pairs of low-pass bucket signals retained the broadband response expected from Huffman-like masks. The pinhole test images were a critical test of the mask practical functionality, as, with the pinhole illuminating only a tiny fraction of the area of the bucket detector, the image signal-to-noise is at a minimum.

The bucket images obtained using these fabricated masks on simple binary and stepped gray-level objects yielded realistic reconstructed high-resolution images of these objects after deconvolving the bucket images using the ideal integer arrays. This again confirms that the masks, as fabricated and imaged by the X-ray bucket signals, were able to replicate and conserve the delta-like properties of the Huffman-like arrays as designed.

Future work may consider applying similar masks to structure broad X-ray beams for X-ray fluorescence imaging applications and other existing scanning probe techniques.

## 9. Related literature

The following reference, not cited in the main body of the paper, has been cited in the supporting information: Arhatari *et al.* (2023 ▸).

## Supplementary Material

Supporting information. DOI: 10.1107/S1600577525002127/mo5298sup1.pdf

## Figures and Tables

**Table 1:** Example metrics for four 11 × 11 arrays The first 2D array is built from the 1D integer Huffman sequence *H*_11_. The second array, 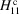, is a compressed integer range Huffman-like version built from the first array. The third is a 2D *B*_11_ array, built from a length 11 binary Barker sequence using an outer product. Barker sequences have unit-magnitude integer elements (*i.e.* they are ‘binary’), with aperiodic auto-correlation values all ≤1. The longest known Barker sequence has 13 elements. Infinite families of Barker-like sequences with merit factors *M*^*f*^ > 6.34 can be constructed (Borwein *et al.*, 2004 ▸). Recent stochastic algorithms have been designed to discover and optimize new such sequences (Bošković *et al.*, 2017 ▸; Bošković & Brest, 2024 ▸). The fourth is *L*_11_, built from the length 11 binary periodic ‘perfect’ Legendre sequence (Golay, 1983 ▸; Hoholdt & Jensen, 1988 ▸; Petersen *et al.*, 2024 ▸), circularly shifted to maximize the aperiodic metrics *R*^0^ and *M*^*f*^.

Type	Range	RMS	MAV	*f* ^*z*^	*M* ^*f*^	*R* ^*0*^	*d* ^*F*^	κ
*H*_11_	±36	11.18	7.94	0/121	3782	123.0	0.033	1.02
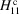	±3	1.52	1.30	11/121	18.24	35.13	0.635	1.31
*B*_11_	±1	1.00	1.00	0/121	5.81	11.00	1.26	2.00
*L*_11_	±1	0.91	0.83	21/121	2.55	5.00	2.09	4.49

**Table 2:** X-ray transmissions and Ta thicknesses for each level of the quaternary Huffman-like masks

Layers/levels	X-ray transmission at 12.4 keV	Ta thickness
0	17.5%	4.8 µm
1	45%	2.2 µm
2	72.5%	0.88 µm
3	100%	0

**Table 3:** Values for the 5 × 5 area around the central peak of the cross-correlation of the 32 × 32 mask X-ray pinhole image with the ideal Huffman-like array (values are given as the percentage of the central peak shown in bold)

−0.4	−1.6	−4.9	−2.0	0.8
0.0	6.6	5.9	0.8	0.2
−3.1	22.6	**100.0**	19.0	−0.4
−0.4	13.2	23.8	0.2	0.1
2.0	4.5	1.1	1.6	0.6
