# Supplementary material for: High-resolution X-ray scanning with a diffuse Huffman-patterned probe to reduce radiation damage
Source: J Synchrotron Radiat. 2025 Apr 9;32(Pt 3):700–17. doi: 10.1107/S1600577525002127 (PMC12067338; doi:10.1107/S1600577525002127)
Supplement: Supplementary file 1 [file s-32-00700-sup1.pdf]

JOURNAL OF SYNCHROTRON RADIATION

Volume 32 (2025)

Supporting information for article:

High-resolution X-ray scanning with a diffuse Huffman-patterned probe to reduce radiation damage

Alaleh Aminzadeh, Andrew M. Kingston, Lindon Roberts, David M. Paganin, Timothy C. Petersen and Imants D. Svalbe

Journal of Synchrotron Radiation

ISSN 1600-5775

Supplementary Material: High-resolution X-ray scanning with a diffuse Huffman-patterned probe to reduce radiation damage

Alaleh Aminzadeh,[a] Andrew M. Kingston,[a,b] Lindon Roberts,[c] David M. Paganin,[d] Timothy C. Petersen[e] and Imants D. Svalbe [d]*

[a]Department of Materials Physics, Research School of Physics, Australian National University, Australia, [b]CTLab: National Centre for Micro- Computed Tomography, Australian National University, Australia, [c]School of Mathematics and Statistics, University of Sydney, Australia, [d]School of Physics and Astronomy, Monash University, Australia, and [e]Monash Centre for Electron Microscopy, Monash University, Australia. Correspondence e-mail: imants.svalbe@monash.edu

© International Union of Crystallography

This Supplementary Material provides additional theoretical background and further technical detail on Huffman sequences and methods to compress their value range to form Huffman-like arrays. It also contains a more extensive description of the practical steps taken to fabricate Huffman-like masks by a precise pixel-wise deposition of patches of tantalum on a silica wafer. These masks were used to modify the transmitted intensity of x-ray beams to have Huffman-like profiles that were used as broad $2D$ scanning probes to image test objects. The diffuse pattern encoded by a Huffman-like intensity profile can be decoded by deconvolution to reconstruct a sharp image. That decoding property is made possible because of the delta-like autocorrelation of Huffman-like arrays. The advantage of scanning objects with a broad Huffman-like x-ray probe is to strongly reduce the rate of local energy deposition and hence minimise radiation damage.

1. General canonical Huffman sequences defined by complex polynomials

Huffman originally derived a sufficient and necessary criterion for constructing complex-valued canonical sequences, for which some integer forms, such as those based upon Lucas-Fibonacci polynomials (Hunt & Ackroyd, 1980; Svalbe *et al.*, 2020), are known. This subsection revises Huffman's construction, without any generalisation.

Huffman defined a canonical sequence as the complex-valued coefficients c_l of a polynomial $P(z) = \sum_{l=0}^{L-1} c_l z^l$, where z_l denotes the lth integer power of a variable z in the complex plane. For a canonical sequence of length $L-1$, Huffman defined a conjugate-reversed or complementary polynomial $Q(z) = \sum_{l=0}^{L-1} \bar{c}_l z^{L-1-l}$, where $\bar{c}_l$ is the complex conjugate of c_l. By construction, the roots of $P(z)$ have the same complex arguments (phase angles) as those of the conjugated $\bar{Q}(z)$ but the magnitudes of their roots are mutually reciprocal (Huffman, 1962). When expressed as a power series, the complex polynomial $P(z)\bar{Q}(z)$ has coefficients pertaining to the autocorrelation values of the sequence c_l.

The canonical condition is that all autocorrelation elements need be zero, except the unavoidable ends with magnitudes $|\bar{c}_0 c_{L-1}|$ and peak value $A_0 = \sum_{l=0}^{L-1} |c_l|^2$ (sum of squared magnitudes). The product $P(z)\bar{Q}(z)$ then collapses to a quadratic in z^{L-1}, which has two roots. Hence all roots of $P(z)\bar{Q}(z)$ must lie on either of two circles centered in the complex plane, with angles pertaining to the $(L-1)^{\text{th}}$ roots of unity, as is true for the roots of $P(z)$ and $\bar{Q}(z)$. Given the aforementioned reciprocal magnitudes between the roots of $P(z)$ and $\bar{Q}(z)$, this in turn means that these roots must occur on a circle of radius R or $1/R$. There are 2^{L-1} choices one can make to place these roots around these circles, at equi-phase angles $\text{Arg}(z_l) = 2\pi l/(L-1)$, which produce (generally complex) Huffman sequences each having the same aperiodic canonical cross correlation, with peak value $A_0 = |\bar{c}_0 c_{L-1}|(R^{L-1} - R^{-(L-1)})$. Hence we may write

$$\mathcal{F}[H_L^s]_q = c_{L-1} \prod_{l=1}^{L-1} (e^{2\pi i q/L} - R^{s_l} e^{2\pi i (l-1)/L}). \qquad (1)$$

The following comments apply in reference to Eq. (1). To numerically construct such a canonical Huffman sequence H of length L with elements c_l, one must choose a fixed radius R (centred on $(0,0)$) for all complex roots z_1 to z_{L-1} and then pick a set of signs $s_l \in \{-1,+1\}$, such that $z_l = R^{s_l} \exp(2\pi i l/(L-1))$. Real-valued c_l are readily fixed by defining z_l in the upper-half of the complex plane to have matching conjugated polynomial zeros in the lower half of the complex plane. The inverse discrete Fourier transform in Eq. (1) provides a convenient numerical algorithm for performing this computation. Due to potential underflow or numerical overflow for certain values of R, it is also advantageous to evaluate an exponential for a sum over logarithms, rather than to compute the series product directly. Since Eq. (1) represents a conversion between a set of polynomial roots and coefficients, the same generic computation can be used for non-canonical Huffman-like sequences, where the roots violate Huffman's criteria (for example when $|s_l| \neq 1$).

2. Correlation and Fourier properties of Huffman sequences

This section provides some examples of, and general properties for, particular integer Huffman sequences.

Integer values for the $1D$ Huffman sequence H_{15} based on Lucas/Fibonacci series are

$$H_{15} = [1, 2, 2, 4, 6, 10, 16, -3, -16, 10, -6, 4, -2, 2, -1],$$

as plotted in Fig. 1. The autocorrelation, $H_{15} \otimes H_{15}$, of length $2 \times 15 - 1 = 29$, is

$$H_{15} \otimes H_{15} = [-1, 0, 0, \cdots, 0, 843, 0, \cdots, 0, 0, -1],$$

which is as delta-like as possible under aperiodic conditions.

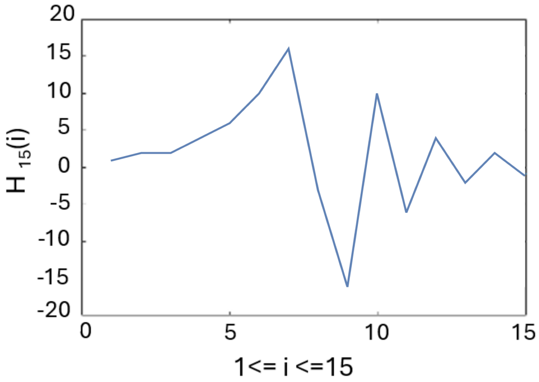

Figure 1
Canonical Huffman length $L = 15$ integer sequence, H_{15}. The sequence values vary between ± 16.

Note the reflected left/right symmetry of the absolute values of the elements about the central element (here -3), with alternating sign changes for all the right elements. Any sequence with this reflected symmetry pattern of element values and signs ensures that every second autocorrelation value will be zero. To obtain zero off-peak autocorrelation values at *all* but the (unavoidable) end elements requires a special choice for the sequence element values. Starting from the second element on the left, note that the next six values are twice the Fibonacci sequence, $2 \times [1, 1, 2, 3, 5, 8]$. Integer sequences built on this Lucas/Fibonacci pattern will have the maximum value as given by Eq.(4) in the main text, which is

$$max(|H_L|) = \lfloor (2/\sqrt{5}) \times \phi^{(L-3)/2} \rceil, \qquad (2)$$

where ϕ is the golden ratio $(1 + \sqrt{5})/2$, and $\lfloor r \rceil$ denotes the integer round operation on real value r.

For H_{31} there are 14 Fibonacci terms, giving the Huffman sequence a maximum value of $2 \times 377 = 754$. This result emphasizes why, for practical mask fabrication, strong but effective compression of the Huffman integer sequences is essential. For $2D$ arrays built using outer products, the maximum value for a non-compressed 31×31 integer Huffman array would be 754^2.

The center term of the Lucas/Fibonacci integer sequences is also a Fibonacci term. In general it is always minus half the value of the third Huffman term before the center. For our example H_{15}, the center term is $-(6/2) = -3$.

In general, the sum of any sequence S_L, $\sum_{i=1}^{L} S_L(i)$, when squared, always equals the sum of *all* the autocorrelation values. For Huffman sequences, the autocorrelation is non-zero only for the center term A_0 and the two end values. The autocorrelation (central) peak value A_0 is always the sum of all squared sequence values, $A_0 = \sum_{i=1}^{L} S_L(i)^2$. For Lucas/Fibonacci sequences, each end of the autocorrelation contributes an off-peak value of $-1 \times +1 = -1$. Then, consistent with Parseval's Theorem:

$$\sum_{i=1}^{L} H_L(i)^2 = \left[\sum_{i=1}^{L} H_L(i) \right]^2 + 2.$$

For our example H_{15} the sequence sum is 29 and $29^2 = 841$. The autocorrelation peak for H_{15} has $A_0 = 843$.

The sequence sums also provide assurance that, after some range compression is applied, the compressed Huffman sequence H_L^c remains closely Huffman-like, as then

$$\left[\sum_{i=1}^{L} H_L^c(i) \right]^2 \approx \sum_{i=1}^{L} H_L^c(i)^2.$$

Not all integer Huffman sequences follow the Lucas/Fibonacci form. For example, at length $L = 11$ there are twin sequences

$$H_{11} = [1, 1, 2, 4, 6, -1, -6, 4, -2, 2, -1],$$
$$H_{11,\text{twin}} = [1, 1, 3, 4, 2, 6, -7, -1, 2, 1, -1],$$

for which, despite slightly different dynamic ranges, all the autocorrelation metrics are exactly the same (as is their aperiodic condition number, $\kappa = 1.0082$). Another example of a different (but less practically useful) form of integer Huffman sequences is

$$H_5 = [27, 72, -24, 8, -3],$$

with autocorrelation

$$[-81, 0, 0, 0, 6562, 0, 0, 0, -81].$$

This sequence has sum 80. Note that $80^2 + 2 \times 81 = 6562$, the autocorrelation peak A_0.

The *periodic* autocorrelation of any length L Huffman sequence H_L, also has length L:

$$(H_l \otimes H_L)_{\text{periodic}} = [0, \cdots, 0, lr, A_0, rl, 0, \cdots, 0].$$

Now the product of the end terms (l, r) of the sequence H_L occurs in the autocorrelation at periodic shifts ± 1, with all the remaining cross-product terms summing to zero, as for the aperiodic case. The shape of the periodic and aperiodic (zero-padded for the aperiodic case) Fourier amplitudes, as shown for H_{15} plotted in Fig. 2, are characteristic for all real Huffman sequences. The Fourier convolution theorem means the coefficients of $\mathcal{F}(H_L \otimes H_L)$ scale as $|\mathcal{F}(H_L)|^2$. The delta-like autocorrelation of $(H_L \otimes H_L)_{periodic}$ means there are only three terms that contribute to the periodic Fourier power spectrum: Fourier frequency $q = 0$ via the autocorrelation peak A_0, and from frequencies $q = \pm 1$, arising from the product lr of both end

elements. For any real Huffman sequence, the inverted, single-period cosine shape for the *periodic* Fourier spectral amplitude coefficients arises from the $q = \pm 1$ terms that are added to the constant (mean value) contribution $q = 0$ from $\sqrt{A_0}$. The magnitude of the Fourier coefficients of H_L, for frequencies $q = 0, ..., L-1$, can then be written as

$$F(q) = \sum_{l=0}^{L-1} H_L(l) + \frac{|lr|}{\sqrt{A_0}}(1 - \cos(2\pi q/L). \quad (3)$$

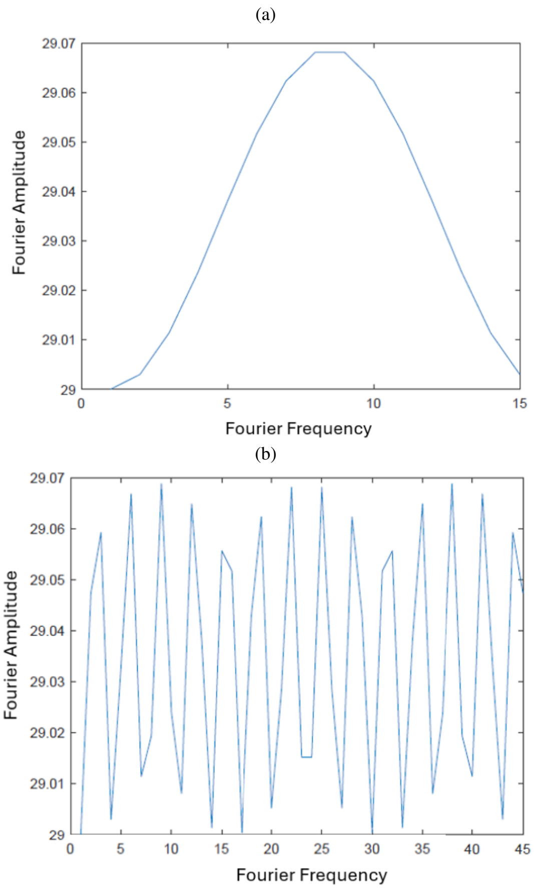

Figure 2
(a) Amplitude of FFT(H_{15}) for the periodic array. (b) Amplitude of FFT(H_{15}) for the aperiodic (zero-padded) array. The horizontal axis shows the Fourier frequency, $0-15$ for the periodic case, $0-45$ for the aperiodic case. The vertical axis shows the Fourier amplitude. The Fourier flatness $d_F = (29.07 - 29.00)/29.03 = 0.0024$ is the same for (a) and (b).

As $\sum_{l=0}^{L-1} H_L(l)$ and $A_0 = \sum_{l=0}^{L-1} [H_L(l)]^2$ are both large relative to lr, the value for d_F, the Fourier flatness for Huffman sequences, is always close to zero (and hence any Huffman sequence autocorrelation is strongly delta-like, even for short sequences).

Note that, for any $2D$ Huffman array built from the outer product of a $1D$ sequence, the value of its $2D$ aperioidic autocorrelation peak, A_0, equals the *square* of the peak autocorrelation value for the $1D$ sequence. Placed along each of the four edges of the autocorrelation are negated copies of the $1D$ autocorrelation (that arise when the left/right and top/bottom edges of the array touch). All other off-peak autocorrelation values are zero. The autocorrelation for $3D$ Huffman arrays has the $2D$ correlation pattern on each of the six cube faces, with its A_0 values the cube of the $1D$ value (and all internal off-peak values being zero).

Table 1 shows that the aperiodic autocorrelation of the $2D$ array built from the $1D$ integer Huffman sequence H_7 is also optimally delta-like. In general, for Huffman sequence H_L, the metric value R^0 is the same in $1D$ as $2D$; the metric M^f for $2D$ is always about half the $1D$ value. For $H_7, R^0 = 18$ for both $1D$ and $2D$ versions, whilst $M^f = 324/2$ in $1D$ and $\approx 324/4$ in $2D$.

Table 1
The 2D aperiodic autocorrelation built from integer Huffman sequence H_7 is optimally delta-like.

1	0	0	0	0	0	-18	0	0	0	0	0	1
0	0	0	0	0	0	0	0	0	0	0	0	0
0	0	0	0	0	0	0	0	0	0	0	0	0
0	0	0	0	0	0	0	0	0	0	0	0	0
0	0	0	0	0	0	0	0	0	0	0	0	0
0	0	0	0	0	0	0	0	0	0	0	0	0
-18	0	0	0	0	0	324	0	0	0	0	0	-18
0	0	0	0	0	0	0	0	0	0	0	0	0
0	0	0	0	0	0	0	0	0	0	0	0	0
0	0	0	0	0	0	0	0	0	0	0	0	0
0	0	0	0	0	0	0	0	0	0	0	0	0
0	0	0	0	0	0	0	0	0	0	0	0	0
1	0	0	0	0	0	-18	0	0	0	0	0	1

3. Generalised reconstruction of binarized Huffman arrays

The array transpose $\intercal$ required to implement the separable scheme for binary sub-element arrays implies a slight modification to the de-correlation of Huffman array implementations as given in the main paper, namely

$$S_T = S_p - S_n = O \circledast P - O \circledast N = O \circledast H, \quad (4)$$

in that each constituent sub-pixel array must be separately transposed in the de-correlating Huffman array (so that any pair of Se_c and Se_d is mutually geometrically orthogonal). For Huffman arrays defined by outer-products of Huffman sequences, one need only define the de-correlating Huffman array H by a transpose of the entire array $H^\intercal$, as this is then the same as transposing each sub-element array. Denoting the transpose of individual sub-element arrays using a subscript $\intercal$, we can summarise the de-correlation as a minor variation on Eq. (4).

Suppose two signals for positive and negative masks Sb_p and Sb_n are acquired with binary arrays B_p and B_n, such that sub-element arrays Se comprising $B_p - B_n$ correspond to elements of the Huffman array H. Denoting these measurements of a desired object O as $Sb_p = O \circledast B_p$, $Sb_n = O \circledast B_n$, the binarized equivalent of Eq. (4) is

$$O \approx (Sb_p - Sb_n) \circledast (B_p - B_n)_\intercal, \quad (5)$$

where $(B_p - B_n)_\intercal = (B_p - B_n)^\intercal$ if and only if the corresponding Huffman array H has transpose symmetry (as inherited from a lower-dimensional outer-product construction).

An example sub-element array for binary Huffman design is shown in Fig. 3(a), corresponding to an original Huffman element with value six. A similar such transposed sub-element array required for de-correlation pertaining to a Huffman element value of five is shown in Fig. 3(b). The simpler "blocked" design choices for practical fabrication are shown in Fig. 3(c) and Fig. 3(d).

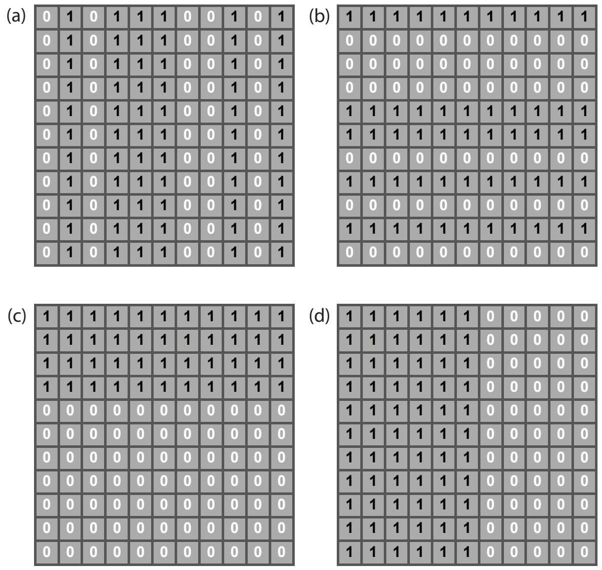

Figure 3
Sub-elements of a $2D$ Huffman array - the black/white ones/zeros represent 100%/0% transmission sub-elements respectively. (a) Randomised sub-element array for an original element value of six. (b) Transpose of randomised sub-element array for decorrelation, for an original element value of five. (c) Contiguous randomised sub-element array for an original element value of four. (d) Contiguous randomised sub-element array for decorrelation, for an original element value of six.

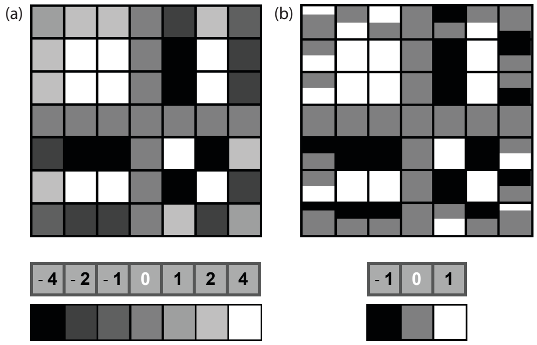

Figure 4
A canonical 7×7 Huffman array binarised. (a) An outer-product of a 7-element Fibonacci based sequence defines the $2D$ canonical Huffman array H, with gray levels in the range depicted by the legend at the bottom. (b) Two binarised arrays subtracted from each other, namely $B_p - B_n$, as a ternary array with identical aperiodic auto-correlation to that of H but which has $4 \times 4 = 16$ times as many elements.

As a complete example of the binarised Huffman transformation, Fig. 4 depicts a canonical 7×7 sized $2D$ Fibonacci Huffman array stemming from an outer product of two identical $1D$ Hunt and Ackroyd (Hunt & Ackroyd, 1980) sequences with 7 elements each. The two signed binarised arrays B_p and B_n, as given in the main paper, have been suitably superposed to represent the Huffman array in the ternary form $B_p - B_n$.

4. Optimising $2D$ Huffman-like arrays using hybrid reverse Monte Carlo

Here we provide an example of a HRMC simulation to optimise a 11×11 Huffman-like array, as per the algorithm outlined in the main paper. With the merit factor M^f as an effective energy term, an example chi-squared χ^2 that incorporates numerous other metrics such as spectral flatness d^F, fraction of zeros f^z, and off-peak correlation ratio R^o can be written as

$$\begin{aligned} \chi^2 = &(M^f - M^f_t)^2/M^f_w + (d^F - d^F_t)^2/d^F_w + \\ &(f^z - f^z_t)^2/f^z_w + (R^o - R^o_t)^2/R^o_w, \end{aligned} \tag{6}$$

where the subscript "t" is the target value and "w" is the weight.

As is typical for reverse Monte Carlo simulation, the individual weights are best empirically chosen. For the Huffman arrays produced in this work, this was done by gradually introducing constraints in several trial runs and checking that the running χ^2 was suitably affected by new each new weight. Similar to topological constraints for atomic systems (Opletal *et al.*, 2013), certain targets such as the fraction of zeros f^z require comparatively strong weights (small weighting factors) in order to compete with all other target values. Interestingly trial simulations displayed "cheating" behaviour for f^z, whereby zeros would be concentrated at the borders, thereby emulating a smaller $2D$ Huffman array for which all other target metrics could be more readily achieved. To circumvent this issue, f^z was measured with respect to the interior of the $2D$ array (with borders 20% of the length).

Figure 5 shows an example HRMC simulation for an 11×11 Huffman array, for integer elements ranging from -3 to +3. During the simulated annealing stage, the dimensionless temperature was linearly ramped from an initial 300 value down to 20 at the mid-point of the x-axis (step number 3×10^7), where-after it was held at 20 until the end of the simulation. For the simplicity of presentation for this particular example, the Monte Carlo step size was not weighted by kT, nor was the range of integer values. Similarly, unlike the HRMC simulations for the Huffman-like arrays used in experiment, the maximum gray level was maintained at value 3 for the entire simulation (i.e. the degree of quantising was not gradually changed as another form of simulated annealing).

Auto-correlation quality metrics including the spectral flatness, merit factor and off peak ratio were optimised to their dimensionless target values of 0.8, 15, and 24 respectively, while other metrics were not optimised. At Monte Carlo step number 6×10^7 (1200 on the scaled x-axis), these appear respectively as the lowest, second lowest and second highest fluctuating curves. The highest such curve in Fig. 5 is the fraction of zero values in the $2D$ array, which was optimised to a target value of 0.25.

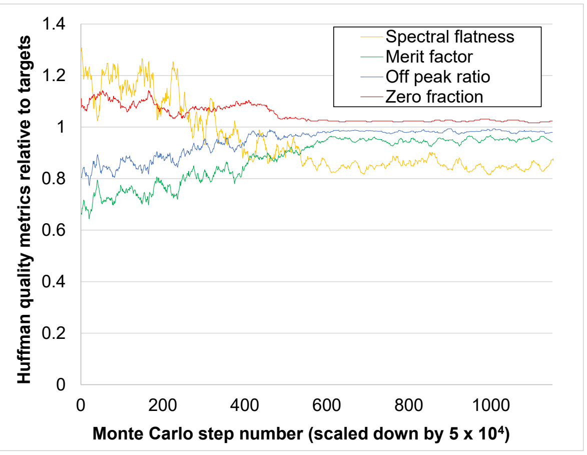

Figure 5
Hybrid reverse Monte Carlo calculation to synthesize a $2D$ Huffman-like 11×11 array with integer-valued elements in $[-3, -2, -1, 0, 1, 2, 3]$. All quality metrics shown have been normalised by their target values, such that a successful simulation would show all graphs meeting with unity values at the final Monte Carlo step.

As evident in Fig. 5, all quantities eventually met their target values, tending toward unity, with the exception of the spectral flatness d^F. This metric was flatter than was thought possible, leading to a more canonical Huffman array. For small $2D$ arrays such as this example, it is possible that only certain sets of metrics are commensurate and it is an open question as to how much these can be simultaneously optimised. The final (randomly fluctuating) relaxed coefficients of the complex polynomial for this simulation is the Huffman-like sequence

$$t_{11} = [0.5143, 0.6039, 0.2587, 0.3291, 1.3959, 1.3873, -0.7717, -1.2099, 0.8503, -1.1455, 1.0000]. \quad (7)$$

The $2D$ HRMC Huffman example from which the performance metrics were computed was given by the integer rounded outer product of t_{11}.

5. Image reconstruction: deconvolution and deblurring

Here we describe further optimisation processing that helped to fine-tune the performance of Huffman-like arrays as the range of element values was compressed relative to the original Huffman array. Arrays that have fewer and smaller off-peak autocorrelation values have better autocorrelation metrics but also yield smaller image reconstruction errors (such as the mean squared error per pixel) when applied to arbitrary test data.

The 2D and 3D Huffman-like masks, H, were tested in computer-simulated conditions by convolving digital test data, I, with the designed Huffman-like array imprints to produce "bucket images", $B(x, y, z) = I \otimes H$, where each bucket value sums all of the intensities that fell under the beam footprint for each translation (x, y, z) across the test data (including an over-scan region that extends beyond the object borders to allow for coverage by the finite size of the beam probe).

The images are reconstructed by cross-correlating their bucket image B with the Huffman-like mask H_T. The off-peak autocorrelation values are known to be small (relative to the correlation peak) and sparse (i.e. there are many autocorrelation zeros). A simple deblurring can be achieved by iterative subtraction of the known (and unwanted) contribution of each off-peak autocorrelation contribution to the original deconvolved image. A scaled and shifted copy of the reconstructed image is subtracted for each non-zero off-peak autocorrelation location. This deblurring procedure also works to reduce the image reconstruction errors that arise from the unavoidable end or edge contributions of the canonical Huffman sequences and arrays.

5.1. Deblured image reconstruction after $2D$ Huffman-like deconvolution

For the best-performing 11×11 Huffman-like masks with element range ± 3, convolved test data were able to be deconvolved and deblurred to reconstruct images of the test data with mean absolute errors of less than 1 gray level per pixel for 8-bit test data. The data ranged from 191×191 flat disc images to high contrast pictorial image data with near-flat discrete Fourier coefficients.

The better masks showed deblurring that converged to a stable mean error (of 0.1 gray levels per pixel that remained near-constant after 10 and further deblur cycles). Some larger masks had reconstruction errors that converged to a minimum after a few deblur cycles but that slowly worsened with further deblurring.

Interestingly, whilst the image reconstruction errors were smaller for arrays with better correlation metrics, that was not strictly always the case for all test data. The transpose symmetric arrays also perform slightly differently on the same test data when rotated by 90°.

The results of image reconstruction from data generated by convolution with an 11×11 Huffman-like array are presented in Fig. 6. For this 2D example, a 191×191 pixel subset of the "Barbara" image is used as the input image. The reconstruction process involves deconvolution and iterative deblurring. Improvement in the quality of reconstructed images over the first 10 deblurring cycles is presented in Table 2.

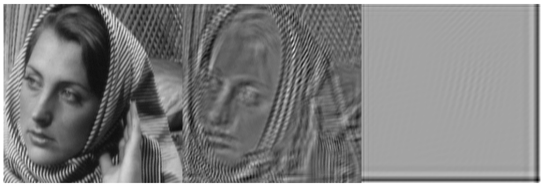

Figure 6
Left: Original 191×191 portion of 8-bit "Barbara" data (zero-padded before array convolution). Centre: deconvolved image reconstruction errors, 0 deblur cycles. Right: image reconstruction errors, 10 deblur cycles. All images shown as max = white = 255, min = black = 0. For actual error image values, see the entries for 0 and 10 deblur cycles in Table 2.

Table 2
191 × 191 pixel image reconstruction errors after convolution of data by Huffman-like 11 × 11 mask and applying *D* deblur cycles.The error measures are, left to right, mean absolute, maximum, minimum, and mean values.

D	*mabs*	*max*	*min*	dc error
**0**	**5.1176**	**35.0819**	**-28.5089**	**2.8642**
1	1.5412	10.7602	-11.3396	-0.1160
2	0.6741	5.9158	-5.3461	0.0146
3	0.3636	3.1774	-2.8311	0.0172
4	0.2280	1.7656	-2.2120	0.0242
5	0.1606	1.1072	-1.8740	0.0246
6	0.1239	1.3733	-2.1798	0.0255
7	0.1037	1.1051	-2.0187	0.0256
8	0.0930	1.2645	-2.1303	0.0257
9	0.0879	1.1606	-2.0614	0.0257
**10**	**0.0855**	**1.2236**	**-2.1055**	**0.0257**

5.2. Deblurred image reconstruction after $3D$ Huffman-like deconvolution

The results of 3D image (or volume) reconstruction from data generated by convolution with an 11 × 11 × 11 Huffman-like array are presented in Fig. 8. For this 3D example, 11 slices of 11 × 11 pixel subsets of the "Barbara" image are used to synthesize the input volume. The reconstruction process involves deconvolution and iterative deblurring. Improvement in the quality of reconstructed images over the first 5 deblurring cycles is presented in Table 4.

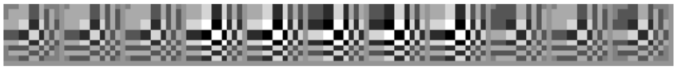

Figure 7
3D Huffman-like array shown as 11 slices, each 11×11 pixels, top to bottom in z. This cube of 1331 voxels has full 3D transpose symmetry. Black = -3, white = +3. Array sum = 35, RMS = 1.65, MAV = 1.48. Autocorrelation metrics: R^o = 20.60, M^f = 12.27, d^f = 0.94, the autocorrelation peak value is 3625, off-peak values range from -176 to +117, 5214 of the 9261 autocorrelation values equal zero, the aperiodic condition number κ = 1.49

Table 3
The aperiodic autocorrelation metrics for each of the 11 (x or y or z axis) 2D planes of the 3D array in Fig. 7. κ = aperiodic slice condition number, tp = 0 if the plane is transpose symmetric, sgn = 0 if slice(i) = slice($12-i$) for i even, and if slice(i) = -slice($12-i$) for i odd.

Slice	R^0	M^f	d^f	κ	tp	sgn
1	12.57	6.19	1.26	1.85	0	0
2	10.67	9.90	0.64	1.60	0	0
3	10.67	9.90	0.64	1.60	0	0
4	19.27	21.41	0.56	1.31	0	0
5	19.27	21.41	0.56	1.31	0	0
6	19.27	21.41	0.56	1.31	0	0
7	19.27	21.41	0.56	1.31	0	0
8	19.27	21.41	0.56	1.31	0	0
9	10.67	9.90	0.64	1.60	0	0
10	10.67	9.90	0.64	1.60	0	0
11	12.57	6.19	1.26	1.85	0	0

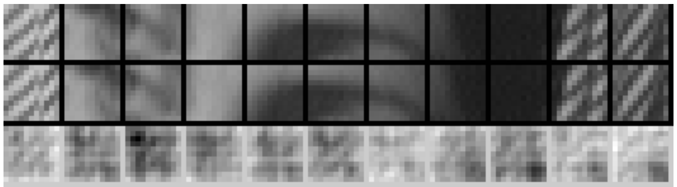

Figure 8
Reconstruction errors for 3D Huffman-like 11 × 11 × 11 array. Top row: 11 slices of 11 × 11 original data, portions of Barbara image. Middle row: the reconstructed image slices from zero-padded data. Bottom row: Image reconstruction errors in each slice after 5 deblur cycles. All images are shown scaled max = white = 255, min = black = 0. Actual reconstruction errors values are printed in Table 4. The smallest reconstruction errors occur here after four deblur cycles.

Table 4
Zero-padded $11 \times 11 \times 11$ data image reconstruction errors after convolution of data by Huffman-like $11 \times 11 \times 11$ mask for the first 5 deblur cycles. The errors are, left to right, the minimum, mean absolute, maximum and mean. Note here the mean absolute error per pixel is least after four deblur cycles.

cycle	*min*	*mabs*	*max*	DC
0	-0.24	24.40	67.25	24.40
1	-1.81	10.40	23.61	10.39
2	-6.49	3.42	10.85	3.01
3	-6.33	1.69	5.86	0.61
4	-7.57	1.48	3.26	-0.95
**5**	**-8.29**	**1.71**	**2.70**	**-1.49**

6. Autocorrelation of binary P and N X-ray images as recombined Huffman-like masks

Validation of the X-ray images taken of the quaternary Huffman-like masks (as P and N mask images combined to form the Huffman-like mask) was presented in the main paper. The multi-level transmission required for the quaternary masks was more difficult to fabricate. This section presents X-ray image validation results for the larger but much simpler to fabricate binary masks.

The images obtained from the $[P, N/N, P]$ mask regions were used to reassemble an image of the signed Huffman-like 15×15 mask. The autocorrelation of the reassembled mask image, shown in Fig.9, shows that the reassembled mask has retained its delta-like property.

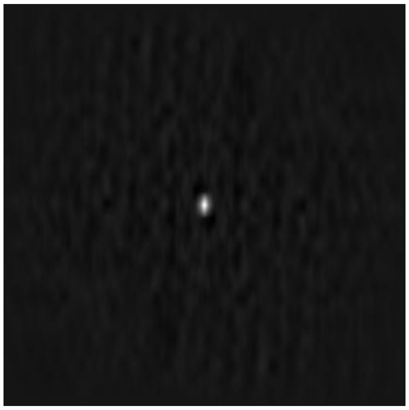

Figure 9
Image of the autocorrelation of the fabricated binary 15 × 15 mask. Note the peak shape is wider vertically than horizontally. Small, similarly elongated off-peak entries are also visible scattered across the image.

Figure 10 shows the autocorrelations of the four detector images obtained for the two 11 × 11 and the two 15 × 15 reassembled masks. The fabricated P and N masks are both intrinsically low-pass filters. Composed as $P - N$, the masks become broad-band filters with autocorelations that are delta-like. The detector images formed by the projected X-ray mask P and N regions had sizes 35×35, 70×70, 49×49, 94×94, hence the autocorrelation peak widths for those images are slightly broader than the single pixel width expected for 11 × 11 and 15 × 15 arrays.

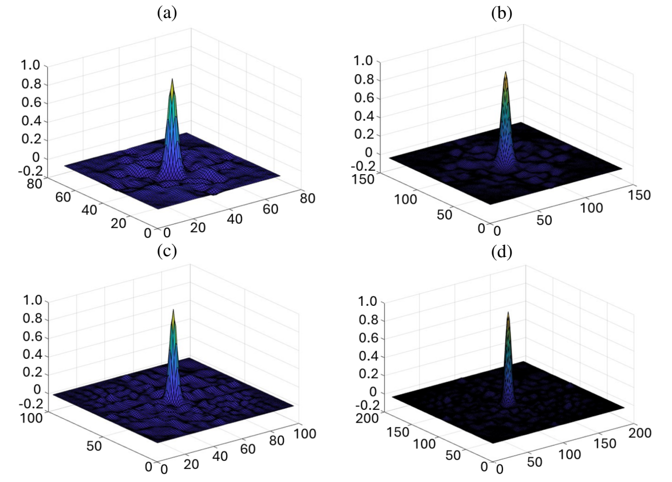

Figure 10
Normalised surface plots for the autocorrelations of detector-measured X-ray images of the binary fabricated masks: (a) 11 × 11 ± 3 gray-levels, 10 micron pixels, (b) 20 micron pixels. Real mask images 15 × 15 ± 3 gray-levels with (c) 10 micron pixels and (d) for 20 micron pixels. The vertical scales have been normalised to value 1.

7. Mask fabrication

The binary mask design consists of transparent and opaque parts. In terms of fabrication, the transparent part is the substrate, which can be glass or SiO_2, while the opaque part is made of a material that is sputtered and patterned onto the substrate. Ideally the sputtered material should be thick enough to block almost all the X-rays at a specific photon energy. However, there are practical limitations of the sputtering process itself. The maximum achievable film thickness using the sputtering technique is approximately 4 μm to 5 μm, which remains challenging. The reason is that such a thick film experiences a high level of stress that can result in cracking, delamination, or other mechanical issues. In addition, there are other issues such as non-uniformity, target erosion, and process instability as the thickness increases to more than a few microns. Thus, the maximum thickness of the mask material is limited to 5 μm.

To select a suitable mask material, a study was conducted to calculate X-ray transmission through 5 μm of various materials available for the sputtering tool at the Research and Prototype Foundry (RPF) at The University of Sydney, Australia, where the masks were fabricated. This analysis covered a range of photon energies from 11 keV to 25 keV as shown in Fig. 11. The available materials included Al, Ti, Ta, Si, Nb, Ru, WTi, and Si_3N_4. Based on the X-ray transmission plot in Fig. 11, WTi and Ta were selected as appropriate materials because of having relatively low X-ray transmissions over the selected photon energies. Among these two materials, Ta was chosen as the material for sputtering purposes due to two main reasons: firstly, Ta films tend to offer better uniformity and adhesion compared to WTi films, especially at thicker deposition levels. Secondly, optimising sputtering parameters and conditions are generally straightforward for a single material like Ta compared to complex alloy compositions like WTi.

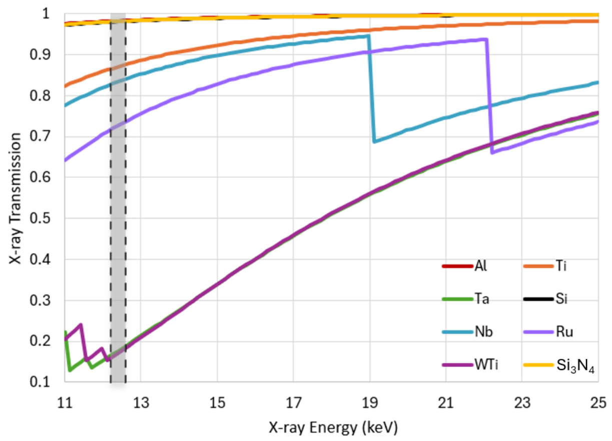

Figure 11
X-ray transmissions through 5 μm thickness of selected materials at a range of photon energies from 11 keV to 25 keV. The gray area between the dashed lines shows the 3% energy bandpass at the selected energy of 12.4 keV.

As shown in Fig. 11, a 5 μm thickness of Ta has a minimum transmission of approximately 17.5% at 12.4 keV photon energy. Note that 12.4 keV was selected rather than 12 keV to avoid the L edge of the Ta and to consider the energy bandpass of 3% at the Micro-Computed Tomography (MCT) beamline (Arhatari *et al.*, 2023) of the Australian Synchrotron where the experiments were performed. The final step before starting

the fabrication process was to choose a substrate, which acts as the transparent parts of the masks. SiO_2 was selected as the substrate due to its widespread availability and common usage. Additionally, its transmission under 12.4 keV is higher than that of Si, another commonly used substrate. The higher transmission of the substrate can improve the contrast of the acquired experimental images.

7.1. Binary masks

Binary masks were fabricated in six steps as schematically depicted in Fig. 12. First, a 6-inch SiO_2 wafer, the substrate, was cleaned with acetone and isopropanol (IPA). Then, the wafer was placed in the sputtering chamber for the sputtering process using an AJA ATC-2200 Sputtering Deposition System. The deposition rate was approximately 8.5 nm per minute. The sputtering time was about 10 hours to achieve nearly 5 μm thick Ta on the SiO_2 wafer. A low deposition rate was necessary to minimise the film stress. After the sputtering process, the 6-inch wafer was coated with a photoresist and then cut into 2 cm $\times$ 2 cm pieces using a Dicing Saw (ADT) machine. The photoresist was used to protect the Ta layer during the dicing process, and it was removed (with acetone) afterwards. The rest of the fabrication process was conducted on individual 2cm $\times$ 2cm pieces.

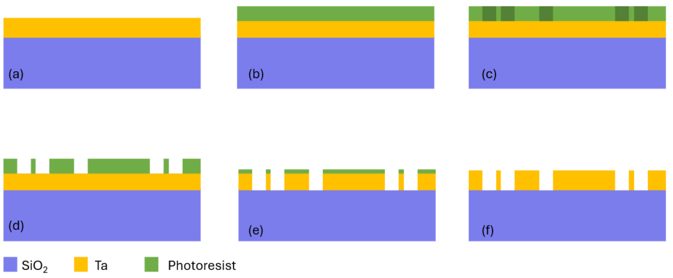

Figure 12
Schematic of the fabrication process for the binary masks: (a) sputtering a tantalum layer on the SiO_2 substrate, (b) spin-coating a photoresist, (c) writing the mask patterns onto the photoresist layer, (d) developing the photoresist, (e) etching tantalum, and (f) removing the remaining photoresist.

After preparing the Ta pieces (see Fig. 12(a)), a lithography process was applied in three steps: 1- spin-coating a photoresist onto the substrate (Fig. 12(b)), 2- patterning (Fig. 12(c)), and 3- developing the photoresist (Fig. 12(d)). Ma-P 1275G (Micro resist technology) was selected as the photoresist because it provides a relatively thick layer (more than 3 μm), which is required for the subsequent etching process. The photoresist was spin coated onto the Ta layer at the maximum velocity of 3000 rpm for 50 seconds and then baked at 105 °C for 120 seconds. At this stage the wafer was ready for patterning. A maskless aligner (Heidelberg MLA100) was used to write binary Huffman-like patterns into the wafer. The dose and the defocus parameters were set to 1000 mJ/cm^2 and 0 respectively. These parameters were chosen from a dose study that we conducted on a few pieces of the same wafer.

Layouts for the binary mask designs used for the maskless aligner are shown in Fig. 13. Figure. 13(a) shows 12 binary masks with four different sizes (i.e. 11 $\times$ 11, 15 $\times$ 15, 32 $\times$ 32, and 43 $\times$ 43) and three resolutions (i.e. 8μm, 10μm, and 15μm). These patterns were written on a 2cm $\times$ 2cm piece of the wafer. The 86$\times$86 binary Huffman-like mask patterns, shown in Fig. 13(b), were written into another piece of wafer. The 86$\times$86 binary mask was also fabricated with 8μm, 10μm, and 15μm resolutions. After writing the patterns, the wafers were developed using an AZ 726 MIF Developer for 7 minutes, followed by rinsing with deionised (DI) water and drying with nitrogen gas. The next step was to etch through the Ta layer using a Reactive Ion Etcher (RIE), as shown in Fig. 12(e). The wafers were placed in the RIE chamber (Plasmatherm Vision) and etched for 50 minutes. This time was sufficient to etch the Ta layer completely and reach to the SiO_2 layer. The RIE recipe was a combination of SF_6, CF_4, CHF_3, and O_2 gases, which we optimised to deep etch the Ta layer. In the last fabrication step, the remaining photoresist was removed by acetone and then the wafer was rinsed and dried by DI water and nitrogen gas respectively (see Fig. 12(f)).

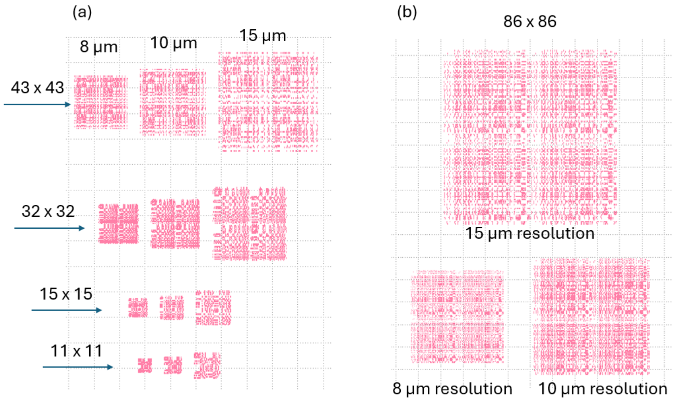

Figure 13
Layout of some of the binary Huffman-like masks with different sizes and resolutions.

7.2. Quaternary masks

Quaternary masks, as explained in the text, have four levels $(0, 1, 2, 3)$. Each level transmits an X-ray beam with steps of increasing intensity. Multiple degrees of X-ray transmission through a mask can be achieved by varying the thickness of the mask material. Pixels at each level of the quaternary mask can be fabricated with a specific thickness to provide the required level of X-ray transmission. As for the binary mask, Ta was chosen as the mask material. Given the maximum achievable thickness of 5 μm using the sputtering technique, as discussed in the previous section, we can estimate the minimum X-ray transmission through our mask. As shown in Fig. 11, the minimum transmission through a 5 μm thickness of Ta is approximately 17.5% at 12.4 keV. This is the transmission through level 0. Based on this minimum transmission, the transmissions through levels 1, 2, and 3 are calculated as 45%, 72.5%, and 100% respectively. Having the X-ray transmissions (T), the thickness of each level can be measured as

$$t = -ln(T)/\mu, \qquad (8)$$

where μ is the linear attenuation coefficient. Using this formula the thicknesses of the levels 1, 2, and 3 were calculated as approximately 2.2 μm, 0.88 μm, and 0 respectively.

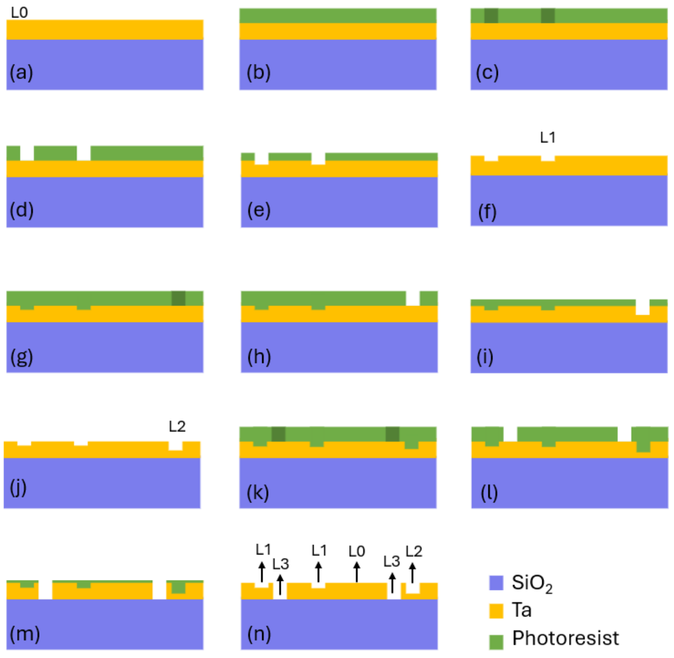

Figure 14
Schematic of the fabrication process for the quaternary masks.(a) Ta film deposition on a SiO_2 substrate. (b)-(d) First lithography process, which includes (b) spin-coating a photoresist on the Ta layer, (c) patterning the photoresist, and (d) developing the photoresist. (e) First etching process to define level one of the quaternary masks. (f) Removing the remaining photoresist. (g)-(h) Second lithography, and (i) etching processes to define level two of the quaternary masks. (j) Photoresist removal. (k)-(l) Third lithography and (m) etching processes to define level three of the quaternary masks. (n) Final result after removing the remaining photoresist.

The fabrication process was a combination of lithography and etching processes similar to the fabrication of the binary masks. However, it was more challenging since multiple lithography steps with precise alignment, and accurate etching time were required to achieve a practical quaternary Huffman-like mask. A schematic of the fabrication process is depicted in Fig. 14. A 4-inch SiO_2 wafer was coated with approximately 5 μm Ta using the sputtering machine and the same sputtering parameters as explained in Sec. 7.1 (see Fig. 14 (a)). Figures 14 (b)-(d) show the first lithography process to define level one of the quaternary Huffman-like masks. The lithography parameters which include type of the photoresist, spin-coating parameters, photoresist backing time, writing parameters as well as the development solution and development time were the same as that for the binary mask. The lithography pattern, however, was different. It contained 15 quaternary masks with five different sizes (i.e. 11, 15, 32, 43, and 86) and three different resolutions (i.e. 10 μm, 15 μm, and 20 μm). The design also included a few test patterns such as circles and bars. KLayout software was used to design the wafer. The pattern for each mask level was drawn in a separate layer to be used for each lithography step. A part of the KLayout design is shown in Fig. 15. Figure 15(a) is layer one (corresponding to level one) of the 15×15 quaternary mask with 20 μm resolution. Layer 2 (corresponding to level 2) and layer 3 (corresponding to level 3) of the same mask are illustrated in Fig. 15 (b) and (c) respectively. Figure 15 (d) is a combination of all layers, which indicate the final result. Note that level 0 has a thickness of approximately 5 μm, which is the thickness of the deposited Ta and can be seen as the background in Fig. 15 (d).

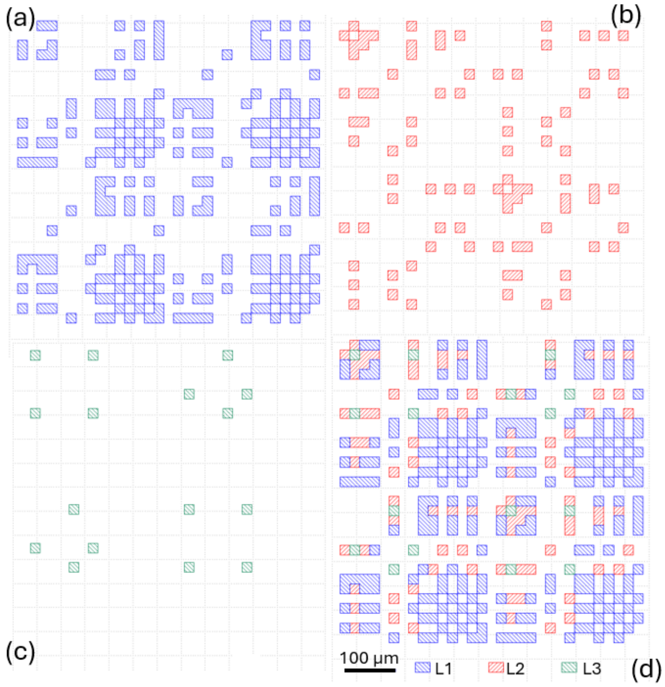

Figure 15
An example of the CAD file for a quaternary mask. Layers 1 (a), layer 2 (b), and layer 3 (c) of the 15 × 15 quaternary mask design with 20 μm resolution. (d) Combination of all layers. The scale bar is for all the images.

After the first lithography step, the wafer was etched (see Fig. 14 (e)) using the RIE machine and the same recipe to deep etch Ta, as discussed in the previous section. However, the etching time was 30 minutes to provide approximately 2.2 μm Ta thickness, which was required for level one of the quaternary masks. The wafer was then washed with acetone to remove the remaining photoresist and rinsed by IPA (see Fig. 14 (f)). At this stage the wafer was ready for the next lithography step, which is shown in Fig. 14 (g)-(h). After spin-coating the photoresist on the etched wafer and the baking process, the patterns in layer 2 of the KLayout design (which correspond to level 2 of the quaternary Huffman-like mask) were written into the photoresist using the mask-less aligner tool. To precisely align the patterns from one layer to the other layer, alignment marks were used in all three layers such that we aligned the alignment marks in layer 2 of the design with the alignment marks in level one, which were written to the wafer in the first lithography step.

After developing the photoresist (see Fig. 14 (h)), the wafer was etched for the second time to define level 2 of the quaternary masks as shown in Fig. 14 (i). The etching time was 43

minutes to achieve the required Ta thickness of approximately 0.88 μm. After the etching process, the remaining photoresist was removed as shown in Fig. 14 (j). Then, the third lithography step was performed to write the third layer (see Fig. 14 (k)-(l)) utilising the same lithography parameters as the previous lithography steps. This was followed by the third etching process. The etching time for level 3 was 60 minutes to remove all the Ta film and reach to the SiO_2 layer as shown in Fig. 14 (m). The last steps of the fabrication process were to remove the remaining photoresist with acetone, then wash and dry the wafer with IPA and nitrogen gas respectively. The result was having 4 levels of Ta thicknesses on a SiO_2 substrate as shown in Fig. 14 (n).

Acknowledgements

AMK and DMP thank the Australian Research Council (ARC) for funding through the Discovery Project: DP210101312. AMK thanks the ARC and Industry partners funding the Industrial Transformation and Training Centre for Multiscale 3D Imaging, Modelling, and Manufacturing: IC180100008. LR thanks the ARC for funding through the Discovery Early Career Researcher Award DE240100006. TP thanks the Australian Research Council (ARC) for funding through the Discovery Project: DP250102966. IDS thanks Andrew Tirkel, Scientific Technology, Brighton, Australia, and Nicolas Normand with Jeanpierre Guedon, Ecole Polytechnique, Nantes, France, for collaboration on designing arrays with strong auto and weak cross correlation. This research was undertaken on the Micro-Computed Tomography beamline at the Australian Synchrotron, part of ANSTO, with beamtime and funding support allocated under grant application 20873. Andrew Stevenson, Benedicta Arhatari and Gary Ruben from MCT made themselves freely available and helpful throughout acquisition of the experimental x-ray imaging data. The authors acknowledge the facilities as well as the scientific and technical assistance of the Research and Prototype Foundry Core Research Facility at the University of Sydney, part of the NSW node of the NCRIS-enabled Australian National Fabrication Facility.

References

Arhatari, B. D., Stevenson, A. W., Thompson, D., Walsh, A., Fiala, T., Ruben, G., Afshar, N., Ozbilgen, S., Feng, T., Mudie, S. *et al.* (2023). *Micro-computed tomography beamline of the Australian Synchrotron: Micron-size spatial resolution x-ray imaging*. *Appl. Sci.* **13**(3), 1317.

Huffman, D. (1962). *The generation of impulse-equivalent pulse trains*. *IRE Trans. Inf. Theory*, **8**(5), 10–16.

Hunt, J. & Ackroyd, M. (1980). *Some integer Huffman sequences (corresp.)*. *IEEE Trans. Inf.* **26**(1), 105–107.

Opletal, G., Petersen, T., Snook, I. & Russo, S. (2013). *HRMC_2.0: Hybrid reverse Monte Carlo method with silicon, carbon and germanium potentials*. *Comput. Phys. Commun.* **184**(8), 1946–1957.

Svalbe, I. D., Paganin, D. M. & Petersen, T. C. (2020). *Sharp computational images from diffuse beams: Factorization of the discrete delta function*. *IEEE Trans. Comput. Imaging*, **6**, 1258–1271.
